# Input from torus longitudinalis drives binocularity and spatial summation in zebrafish optic tectum

**DOI:** 10.1186/s12915-021-01222-x

**Published:** 2022-01-25

**Authors:** Alexander L. Tesmer, Nicholas P. Fields, Estuardo Robles

**Affiliations:** Department of Biological Sciences and Purdue Institute for Integrative Neuroscience, Purdue University, West Lafayette, IN USA

**Keywords:** Zebrafish, Genetic labeling, Pyramidal neuron, Optic tectum, id2b, hspGGFF23c, Atoh7

## Abstract

**Background:**

A continued effort in neuroscience aims to understand the way brain circuits consisting of diverse neuronal types generate complex behavior following sensory input. A common feature of vertebrate visual systems is that lower-order and higher-order visual areas are reciprocally connected. Feedforward projections confer visual responsiveness to higher-order visual neurons while feedback projections likely serve to modulate responses of lower-order visual neurons in a context-dependent manner. Optic tectum is the largest first-order visual brain area in zebrafish and is reciprocally connected with the torus longitudinalis (TL), a second-order visual brain area that does not receive retinal input. A functional role for feedback projections from TL to tectum has not been identified. Here we aim to understand how this feedback contributes to visual processing.

**Results:**

In this study, we demonstrate that TL feedback projections to tectum drive binocular integration and spatial summation in a defined tectal circuit. We performed genetically targeted, cell type-specific functional imaging in tectal pyramidal neurons (PyrNs) and their two input neuron populations: retinal ganglion cells (RGCs) and neurons in TL. We find that PyrNs encode gradual changes in scene luminance using a complement of three distinct response classes that encode different light intensity ranges. Functional imaging of RGC inputs to tectum suggest that these response classes originate in the retina and RGC input specifies PyrN functional classes. In contrast, TL input serves to endow PyrNs with large, compound receptive fields that span both retinal hemifields.

**Conclusions:**

These findings reveal a novel role for the zebrafish TL in driving binocular integration and spatial summation in tectal PyrNs. The neural circuit we describe generates a population of tectal neurons with large receptive fields tailored for detecting changes in the visual scene.

**Supplementary Information:**

The online version contains supplementary material available at 10.1186/s12915-021-01222-x.

## Background

A central question in neuroscience is how complex response properties in the brain arise from sensory inputs with relatively simple response properties. The zebrafish optic tectum is a powerful system to study how the brain processes visual inputs from the retina. Zebrafish are amenable to transgenesis techniques and optically transparent as larvae, enabling noninvasive monitoring of neuronal activity in vivo [1]. The optic tectum is the largest visual area in the zebrafish brain and has been directly implicated in both prey capture and visual escape behavior [2–5]. In the adult zebrafish tectum single neuron recordings previously identified visually responsive neurons with large, compound receptive fields (RFs) [6]. These compound RFs consisted of multiple, non-contiguous regions of visual space. The circuitry that generates this complex response property has not been identified. Does tectum contain neurons that are specifically innervated by RGCs with distant and non-overlapping RFs? Alternatively, do compound RFs in tectum arise via convergent feedback projections from a higher order visual area?

One challenge to constructing cellular-resolution models of tectal function is the extensive cell type diversity in tectum [7, 8]. Cell type-specific transgenics hold the promise of enabling targeted analyses of how distinct cell types contribute to visual processing. We previously identified *id2b:gal4* as a transgenic marker that preferentially labels a cholinergic tectal interneuron, the pyramidal neuron (PyrN) [9]. PyrNs are highly conserved among the ray finned fish and have been described morphologically in adult zebrafish [6], goldfish [10, 11], and perch [12]. Tectal PyrNs have also been identified via intracellular marker injection following electrophysiological recordings of tectal neurons in goldfish [11] and carp [13]. In these electrophysiological studies, PyrNs were the most frequently encountered cell type, suggesting they are among the most numerous neuron types in the teleost tectum. Despite their prevalence, their role in visual processing remains unclear. Our preliminary characterization of PyrN responses to visual stimuli revealed that these neurons exhibit large, compound visual RFs that span both retinal hemifields. We set out to understand the neural circuitry that generates this complex visual response property.

**Fig. 1:**
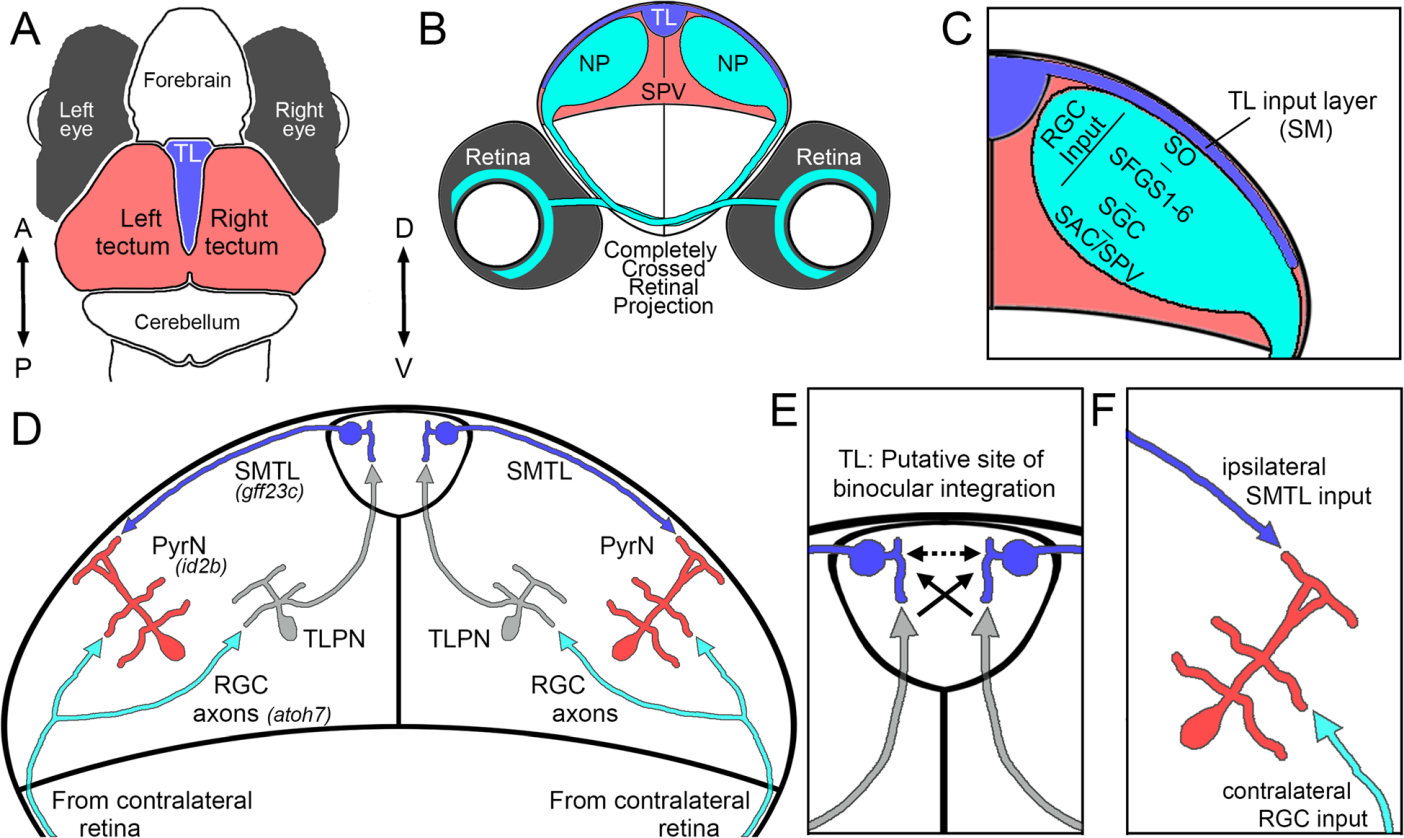
Overview of tectum-TL circuitry and PyrN synaptic input. **A** Schematic dorsal view of the larval zebrafish brain **B**. Schematic coronal view of the larval brain at the level of anterior tectum. Note each lobe of tectum is innervated by TL and contralateral retina. **C** Enlarged coronal view of tectum. Note superficial input layer from TL and deeper neuropil innervated by retinal axons. **D** Putative tectum-TL circuitry. Contralateral retina provides synaptic input to both TLPNs and PyrNs. TLPNs send axonal projections to TL. SM-projecting TL neurons (SMTLs) form axon terminals in tectum that represent a second synaptic input onto PyrNs. **E** Putative circuitry that generates binocular responses in PyrNs. Transfer from one side of TL to the other is likely mediated by both TLPN axons that cross the midline of TL as well as commissural interneurons within TL. **F** PyrNs in tectum receive input from both ipsilateral TL and contralateral retina

PyrNs are known to be innervated by two presynaptic inputs: (1) RGC axons originating from contralateral retina and (2) axons from ipsilateral torus longitudinalis (TL; see Fig. 1) [14, 15]. TL is a second order visual area that forms a feedback projection to tectum [16], but little is known regarding its functional contributions to visual processing. PyrNs have a distinctive morphology characterized by three stratified neurite arbors that target distinct layers of the tectal neuropil: an apical dendrite in stratum marginale (SM), an intermediate dendrite within the stratum fibrosum et griseum superficiale (SFGS), and an axonal arbor within the stratum griseum centrale (SGC) layer of tectum (Fig. 1C, D) [9]. The SM layer of tectum is exclusively innervated by axons from TL [17–21], which form excitatory synapses onto the spines of PyrN apical dendrites [10, 15]. Conversely, the PyrN dendrite located in SFGS receives direct input from RGCs in contralateral retina [15]. Despite its known connectivity, how RGC and TL input contribute to the functional responses of PyrNs has not been directly examined.

Our group recently described the unique wiring geometry between SMTLs and PyrNs in the larval zebrafish [22]. This study used cell type-specific transgenics to examine reconstructions of single PyrNs and their two synaptic inputs: RGC axons and TL axons. These experiments revealed that (1) PyrN dendrite forms small arbors in both SM (TL input) and SFGS (RGC input), (2) axons from TL form extremely large arbors in SM that exhibit a high degree of overlap, and (3) RGC axons in SFGS form small arbors. Based on these anatomical findings, we hypothesized that compound visual RFs in PyrNs arise via spatial summation generated by highly convergent feedback input from TL. To test this, it was first necessary to characterize the visual response properties of PyrNs. Genetically targeted calcium imaging revealed that PyrNs encode gradual changes in luminance with a complement of three response classes: ON responses with peak activity near the maximum intensity, OFF responses with peak activity near the minimum intensity, and DUAL responses that exhibit two peaks—one during the increase and another during the decrease. Functional imaging of RGC inputs to the tectum suggests that ON, OFF, and DUAL responses in PyrNs are specified by input from complementary ON, OFF, and DUAL RGCs. We also discovered that the majority of PyrNs are binocular, responding to visual stimulation of either eye. Functional imaging in TL neurons that project to SM revealed matching ON, OFF, and DUAL response classes and identified TL as the site of binocular integration. Laser ablation of TL confirmed that TL input is necessary for the interocular transfer that drives binocular responses in PyrNs. Together these findings support a model in which RGC input specifies functional PyrN classes (ON, OFF, and DUAL), while convergent input from TL neurons with matching responses endows PyrNs with large, compound RFs. By mediating binocular integration and spatial summation, the TL generates a population of tectal neurons with functional properties tailored to monitoring changes in the visual scene.

## Results

### Tectal pyramidal neurons encode gradual changes in scene luminance

**Fig. 2:**
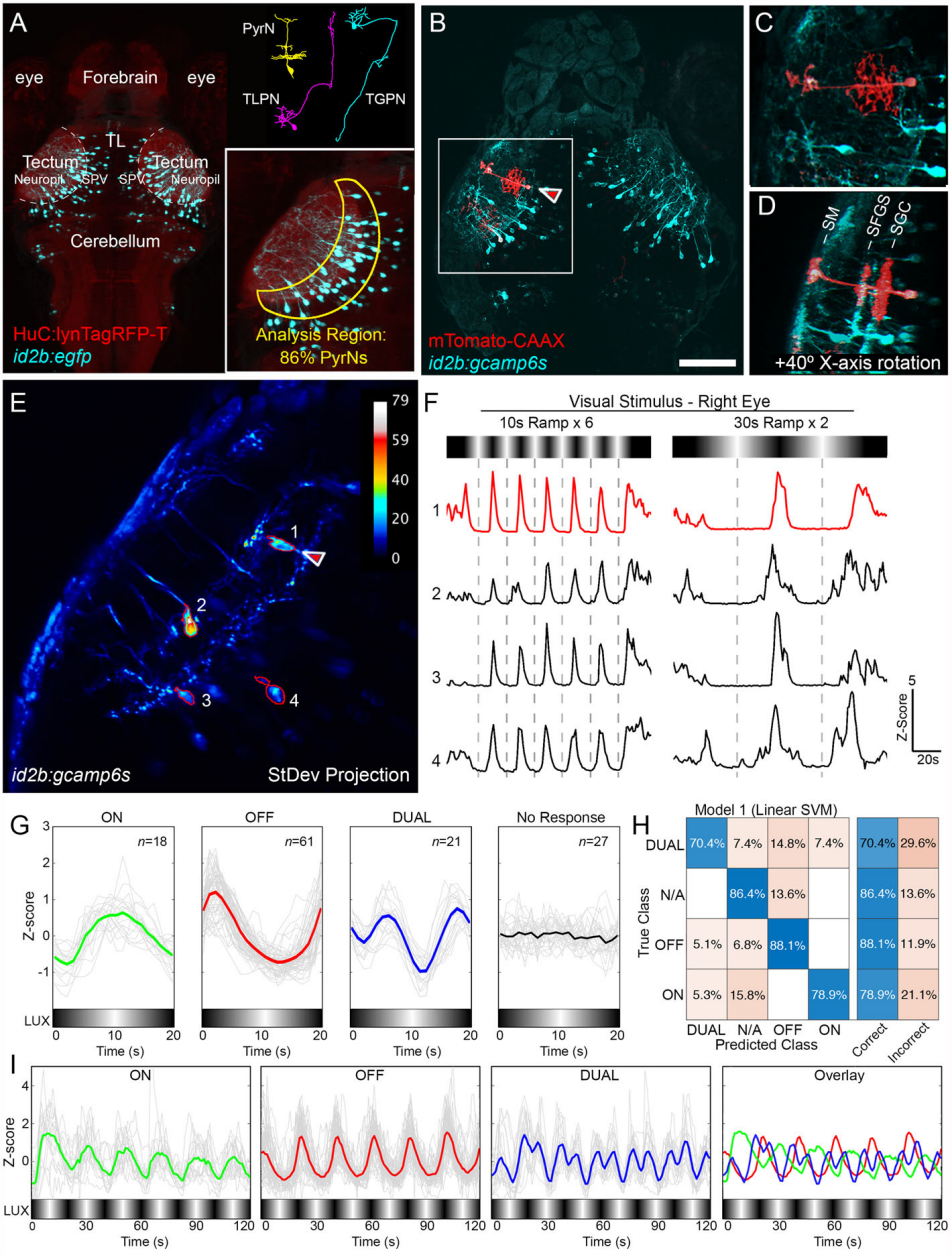
PyrNs respond to gradual changes in display luminance. **A** Overview of expression pattern in a 6 dpf *id2b:gal4,uas:egfp,HuC:lynTagRFP-t* larva. *id2b*-positive neurons are labeled in cyan, while axon tracts and neuropil are labeled in red. Upper right: reconstructions of the three neuron types labeled in the *id2b:gal4* transgenic. Lower right: region of analysis that includes the deep tectal neuropil and shallow SPV. **B** Maximum projection whole-brain image of a 7dpf *id2b:gal4,uas:gcamp6s* larva with a single mTomato-labeled PyrN (red arrowhead). **C** Magnified view of boxed region in **B** containing a mTomato-labeled PyrN. **D** 40° rotated view around *X*-axis of boxed region in **B**. Note three distinct neurite stratifications in SM, SFGS, and SGC layers. **E** Standard deviation (StDev) projection image of a multiphoton timeseries acquired from region indicated in **B** during presentation of 10s ramp stimulus. Pixels with higher values correspond to regions that underwent large variations in fluorescence intensity during recording interval. Red arrowhead indicates mTomato labeled PyrN in **B**–**D**. **F** Ramp-evoked responses in neurons labeled 1–4 in **E** during presentation of 10s and 30s ramp stimuli. Red trace corresponds to the mTomato labeled PyrN in **B**–**E**. Note that all four neurons exhibit strong cycle locked responses that peak near the minimum stimulus light intensity. **G** Output of linear SVM classifier run on data acquired from 127 PyrNs in 6 larvae during presentation of 10s ramp stimulus. Note ON, OFF, and DUAL responses and a subset of neurons that did not respond to visual stimulation. **H** Confusion matrix demonstrating SVM performance compared to investigator-determined “true” classification. **I** Average responses to 10s ramp stimulus recorded from 100 active PyrNs in **G** classified by the linear SVM classifier as ON, OFF, or DUAL. Fourth panel depicts merged overlay of three response types. Note sequential activation of DUAL, ON, and OFF PyrNs during each cycle of the luminance ramp. Scale bar, 50μm in **A**, 75μm in **B**, 30μm in **C**–**D**, and 25μm in **E**. TL Torus longitudinalis, SPV stratum periventriculare

The *id2b:gal4* transgene labels three distinct tectal neuron types: PyrNs, torus longitudinalis projection neurons (TLPNs), and tegmental projection neurons (TGPNs; Fig. 2A) [9]. To quantify the proportions of each cell type in our functional imaging experiments, we injected *uas:mtomato-CAAX* DNA into *Tg*(*id2b:gal4,uas:gcamp6s*) double transgenic embryos. This produced larvae in which GCaMP6s was expressed in all *id2b:gal4*-positive neurons, and the red fluorescent protein mTomato was expressed mosaically, permitting morphological classification of single neurons (Fig. 2B). PyrNs were identified by their distinctive tristratified morphology as well as the lack of an extratectal axon (Fig. 2C, D). The majority of labeled neurons were PyrNs (25 of 34 neurons in 12 larvae, 73.5%), while only small fractions were TLPNs and TGPNs (11.8% and 14.7%, respectively). This analysis also confirmed our previous findings [9] that the majority of PyrN and TLPN cell bodies are located in either the tectal neuropil or the shallow stratum periventriculare (SPV, the main cell body layer; data not shown). This is in contrast to TGPNs, which have cell bodies located in the deep SPV. Based on these different cell body positions, we restricted our analyses to a region spanning the deep neuropil and shallow SPV (Fig. 2A). Based on the relative proportions of PyrNs and TLPNs, we estimate that restricting analysis to *id2b:gal4-*positive cell bodies within this region results in 86% of analyzed cells being PyrNs. Together these findings confirm that the *id2b:gal4* transgenic can be used to effectively target PyrNs of the larval zebrafish tectum.

It had previously been demonstrated by single neuron electrophysiology that PyrN responses exhibit long latencies [11]; therefore, we hypothesized that gradual changes in luminance could be an effective stimulus for PyrNs. To test this, we designed a whole-field luminance ramp stimulus consisting of repeating cycles of a linear increase in display brightness followed by a linear decrease. We utilized ramp times of 10 and 30s, corresponding to 20s and 60s cycle durations. Display luminance was calibrated so that the minimum stimulus intensity had an intensity of 0.4 LUX (same as background light level during adaptation in the imaging chamber) and the maximum stimulus intensity had an intensity of 32 LUX (Fig. 2A). Stimuli were presented to one side of the larva to minimize stimulation of the contralateral eye. Ramp stimulus-evoked responses were initially monitored in morphologically identified PyrNs expressing both GCaMP6s and mTomato (Fig. 2C, D). The activity of nine morphologically identified PyrNs was monitored during presentation of both 10s and 30s ramp stimuli to the contralateral eye. Eight of these neurons exhibited strong responses that were time-locked to the ramp cycle, whereas a single morphologically identified PyrN did not exhibit visual responses (data not shown). Seven of these PyrN responses consisted of single peaks during the troughs of the 10s ramp stimulus, when light intensities were near minimum (Fig. 2E, F). All PyrNs that responded consistently to the 10s ramp stimulus also responded with similar, stimulus-locked responses to the 30s ramp stimulus (37 of 37 PyrNs in 10 larvae; Fig. 2F).

Intracellular recordings from tectal PyrNs in carp and goldfish previously identified a subset of PyrNs responsive to increases in illumination (ON) and a subset responsive to decreases (OFF) [11, 13]. This suggested that PyrNs are comprised of at least two functional classes; however, the number of neurons examined in these studies was insufficient to rule out additional classes. To determine the number of distinct PyrN responses to ramp stimuli, we initially employed *K*-means clustering to classify PyrN responses based on GCaMP6S dynamics relative to stimulus timing. Using the elbow method with the Calinski-Harabasz criterion to determine optimal cluster number, this approach typically identified an optimal cluster number of 6 or 7. However, *K*-means clustering outputs were variable and not consistently reproducible (data not shown). Visual inspection of many *K*-means clustering outputs revealed that these clusters could consistently be grouped into three response classes based on similar kinetics: ON responses had a peak near the maximum intensity of 32 LUX, OFF responses had a peak near the minimum intensity of 0.4 LUX, and DUAL responses exhibited two peaks, one during light intensity increase and the other during decrease (Additional file 1: Figure S1). Within each class (ON, OFF, and DUAL) individual PyrN responses exhibited subtle differences in kinetics; therefore, we cannot exclude the possibility that the classes we describe are comprised of multiple subtypes. To establish an objective classification method for PyrN responses, we then implemented a supervised machine learning approach. A linear support vector machine (SVM) was trained using a set of experimenter-classified data to identify ON, OFF, and DUAL responses (Fig. 2G; Additional file 2: Data S1). Using a 5-fold cross-validation technique, the SVM achieved a mean accuracy of 80.9% on validation datasets using 10s ramp stimuli (Fig. 2H; Additional file 3: Data S2) and 74.4% on datasets using 30s ramp stimuli (data not shown; Additional file 4: Data S3). The average ramp responses of 92 PyrNs classified as ON, OFF, DUAL, or non-responsive by the SVM classifier are shown in Fig. 2G. Figure 2I summarizes how this complement of PyrN responses can encode slow light fluctuations, with one full cycle encoded by an ON-DUAL-OFF-DUAL activation sequence. These findings demonstrate that visually responsive PyrNs are functionally heterogeneous, comprised of three classes active within different light intensity ranges.

Response kinetics within each class were analyzed using automatic peak detection to extract two measurements from ramp-response datasets: (1) the light intensity when GCaMP6s signal reached 50% of the peak value (LUX at half-max) and (2) the duration of the response measured at 50% of peak value (width at half-max). Only peaks with a minimum *Z*-score of 0.75 were included in this analysis. For the DUAL response class, the LUX at half-max analysis used the more prominent OFF peak, whereas the width at half-max analysis included both peaks if they exceeded the peak prominence threshold. The LUX at half-max analysis confirmed that ON, OFF, and DUAL classes responded at significantly different light intensities during 10s ramp presentation (21.14±1.35, 6.57±0.27, and 13.59±0.72 LUX; ±SEM, *p*<0.0001, one-way ANOVA with Tukey’s multiple comparisons test). Width at half-max analysis revealed no significant difference between ON, OFF, and DUAL PyrN responses to 10s ramp stimuli (6.9±0.8, 6.57±0.27, and 5.675±0.53 s; ±SEM, *p*=0.29, one-way ANOVA with Tukey’s multiple comparisons test). It should be noted, however, that our use of a calcium sensor with slow kinetics means that LUX at half-max and width at half-max measurements may not precisely capture the initiation or duration of increased PyrN firing in response to luminance changes. Despite this limitation these data strongly suggest that ON, OFF, and DUAL PyrN classes respond over distinct light intensity ranges.

### A subset of RGCs innervating the tectum encode gradual changes in luminance

**Fig. 3:**
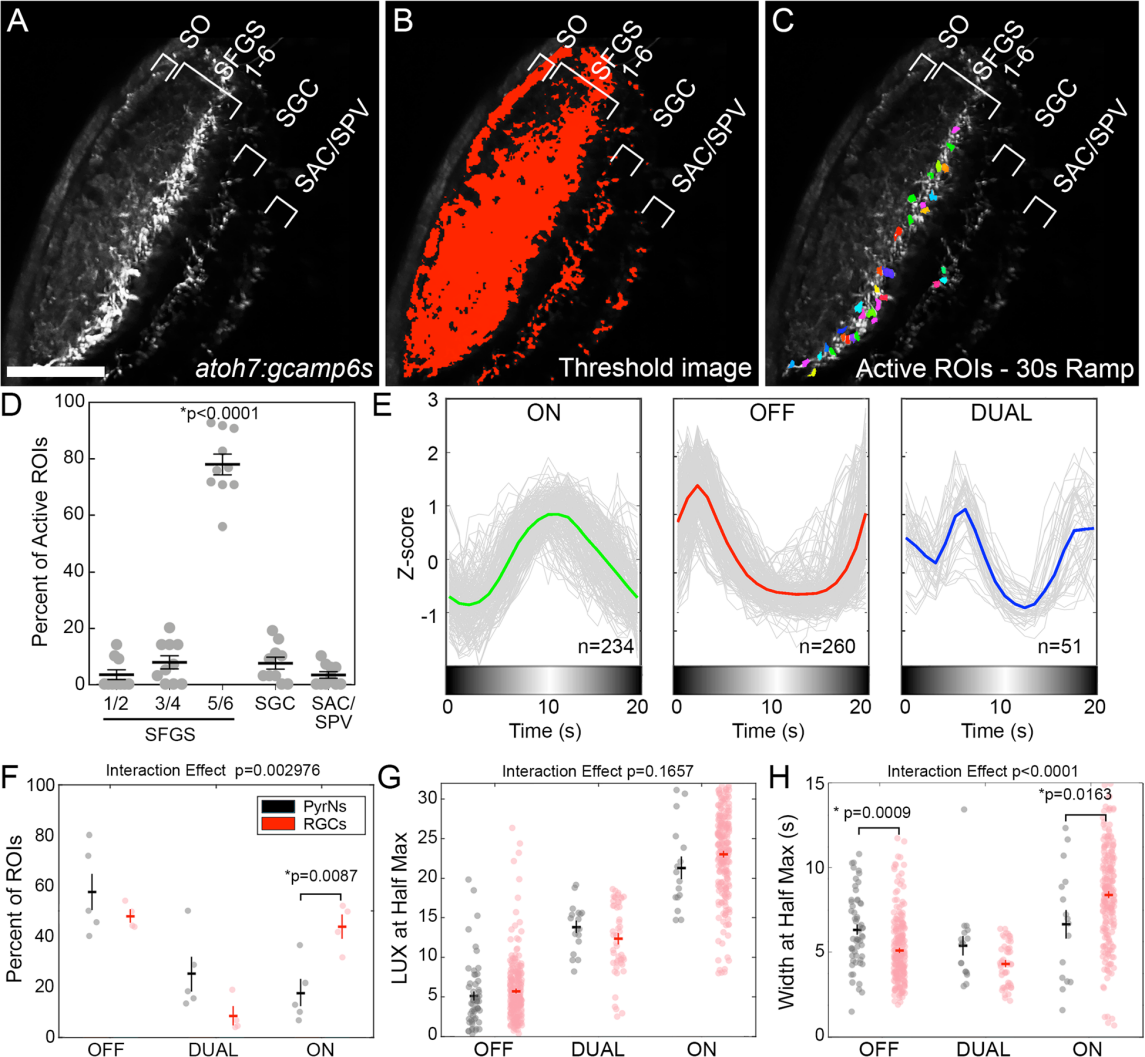
A subset of RGC inputs to tectum encode gradual changes in display luminance. **A** Retinal input layers of the tectal neuropil visualized in the left tectum of a 7dpf *atoh7:gcamp6s* larva. For clarity non-retinal layers are not labeled. **B** Threshold image of GCaMP6s signal in **A**. **C** Image in **A** with colored overlay denoting active ROIs detected during presentation of 30s ramp stimulus. **D** Layer distribution of ramp-responsive RGC ROIs. Data from 367 ROIs detected in 7 larvae. One-way ANOVA with Tukey’s multiple comparisons test was performed, *p* value <0.0001 is for each pairwise comparison between SFGS5/6 and every other group. **E** Average responses of ON, OFF, and DUAL response classes detected by linear SVM trained on PyrN 30s ramp data. Data from 545 ROIs detected in 6 larvae. **F** Comparison of response class distribution between PyrNs and RGC inputs. Note greater proportion of ON responsive units among RGC inputs compared to PyrNs. *N*=30 neurons from 5 larvae, two-way ANOVA, interaction effect *p*=0.002976, with posthoc unpaired *t* test. **G** Comparison of response onset (LUX at half-max) in PyrNs and RGC ROIs during 10s ramp stimulus presentation. Two-way ANOVA, interaction effect *p*=0.1657, with posthoc unpaired *t* test. **H** Comparison of response duration (width at half-max) in PyrNs and RGC ROIs during 10s ramp stimulus presentations. Two-way ANOVA, interaction effect *p*<0.0001, with posthoc unpaired *t* test. Note: for *t* test comparisons in **F**–**H** only *p* values that reached significance are shown. Scale bar: 50μm in **A**–**C**

PyrNs form a dendritic arbor in the SFGS layer that is innervated by RGC axons [15]. Several studies have demonstrated that RGCs responsive to whole-field light steps innervate the SFGS5/6 layers of tectum [5, 23, 24], the same layers in which PyrNs form a dendrite. To determine if the visual response kinetics we observe in PyrNs could be inherited from presynaptic RGCs, we examined ramp-evoked responses in RGC axon terminals using an *atoh7:gal4* transgenic line to drive GCaMP6s expression (Fig. 3A, B) [25]. Automated detection of active ROIs was performed using correlation-based image segmentation [23, 26]. We predicted that many RGCs would respond to rapid changes in illumination, but only a subset would respond to gradual changes. Therefore, we restricted our analysis to retinal inputs that exhibited strong responses to 30s ramp stimuli (Fig. 3C). To calculate the percentage of RGC inputs that responded strongly to 30s luminance ramps, we calculated the active ROI area (sum of color-coded regions in Fig. 3C) relative to the total area of *atoh7:gal4* labeled inputs (thresholded area in Fig. 3B). This analysis confirmed that only a small percentage of RGC inputs exhibit strong responses to 30s ramp stimuli (9.28±2.9%, *n*=8 larvae). The tectal neuropil is a multi-layered structure that contains nine distinct retinal input layers: stratum opticum (SO), six sublayers of the SFGS, SGC, and a thin layer between the stratum album centrale and the SPV (SAC/SPV) [27]. The majority of 30s ramp-responsive ROIs were located within the deep SFGS5/6 sublayers (Fig. 3D) with small numbers of ROIs occasionally detected in SFGS1/2, SFGS3/4, SGC, and SAC/SPV layers. Ramp-responsive ROIs were never detected in SO. Since PyrNs form a stratified dendrite in SFGS5/6, these findings are consistent with ramp-responsive RGCs providing direct synaptic input to PyrNs.

To classify RGC response types, we employed the linear SVM, trained on 10s ramp PyrN data, to classify RGC responses to 10s ramp stimuli (Additional file 5: Data S4). The SVM was able to classify 83.1% of RGC ROIs as ON, OFF, or DUAL (Fig. 3E; 180 ROIs from 4 larvae), and qualitatively, these response classes exhibited response kinetics similar to PyrNs. Quantification of the proportions of ON, OFF, and DUAL response classes revealed that RGCs exhibited a significantly larger proportion of ON responsive ROIs compared to PyrNs (Fig. 3F). Comparison of LUX at half-max values between PyrNs and RGCs revealed no significant difference in responses to either 10s ramps (Fig. 3G) or 30s ramps (Additional file 6: Figure S2). Small but significant differences were detected when comparing width at half-max values between PyrNs and RGCs during 10s ramp stimulus presentation (Fig. 3H). OFF RGC ROIs exhibited shorter response durations (*p*=0.0009, unpaired *t* test), whereas ON RGC ROIs exhibited longer responses (*p*=0.0163, unpaired *t* test). Overall, the similar kinetics of ON, OFF, and DUAL responses in RGCs and PyrNs are consistent with a model in which these response classes originate in the retina. These anatomical and functional data together suggest that PyrN response classes are specified by synaptic input from either ON, OFF, or DUAL RGCs.

### Binocular responses in PyrNs

**Fig. 4:**
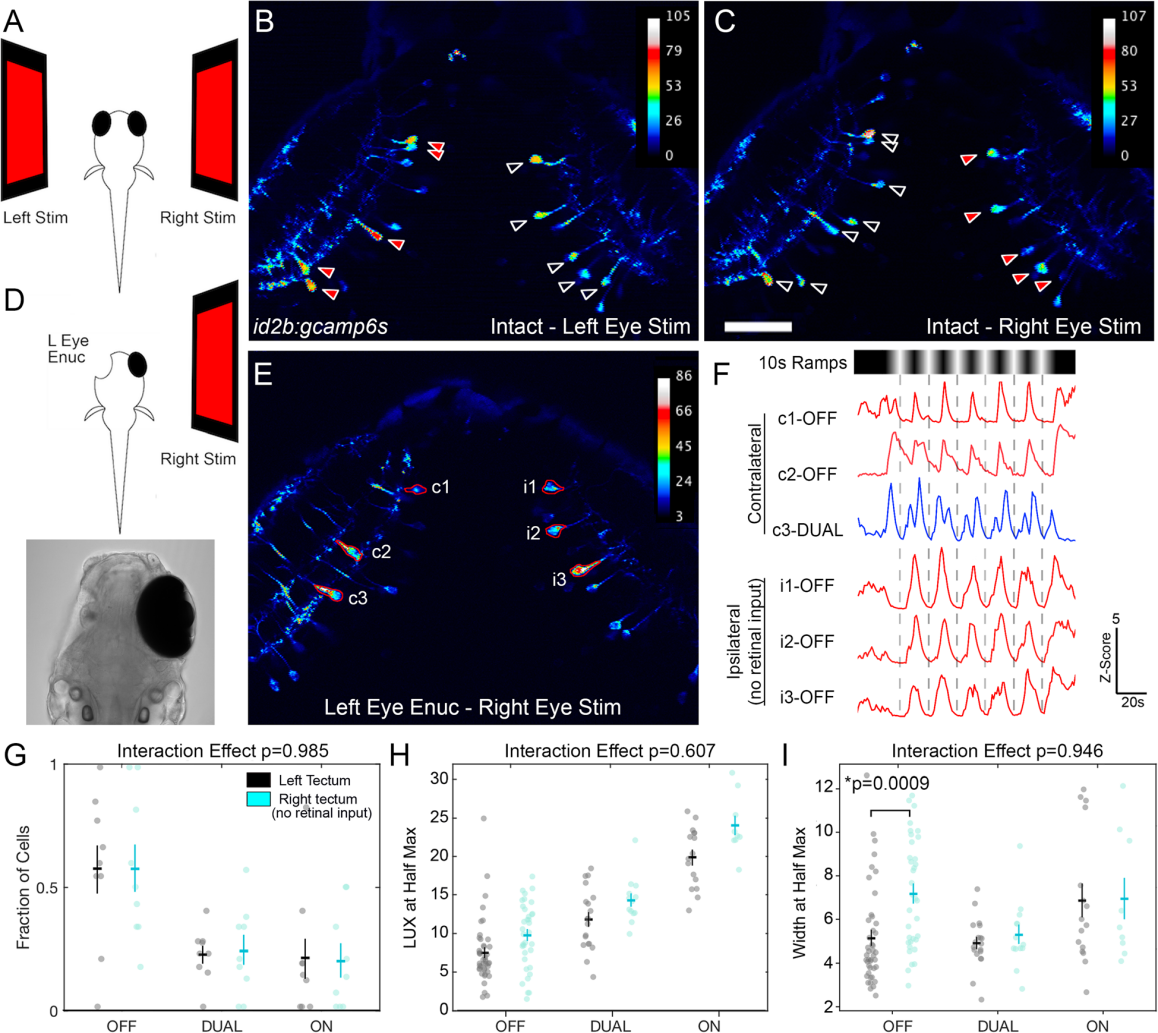
PyrNs exhibit binocular responses. **A** Schematic overview of left and right stimulus presentation experiment. **B** StDev projection image of a timeseries acquired from a *id2b:gcamp6s* larva during ramp stimulus presentation to the left eye. Note the presence of active PyrNs in both the contralateral OT (black arrowheads) and ipsilateral OT (red arrowheads). **C** StDev projection image of same tectum as **B** during 10s ramp stimulus presentation to the right eye. Note the presence of active PyrNs in both the contralateral OT (black arrowheads) and ipsilateral OT (red arrowheads). **D** Schematic overview of stimulus presentation to the right eye of enucleated larvae. Lower panel is a transmitted light image of an 8dpf larva in which the left eye was surgically removed at 3dpf. **E** StDev projection image of a timeseries acquired from a left eye-enucleated larva during 10s ramp stimulus presentation to the right eye. **F** Ramp-evoked responses in contralateral neurons (c1-3) and ipsilateral neurons (i1-3) of tectum shown in **E**. Note that all six neurons exhibit consistent responses to each ramp stimulus cycle. **G** Response class distribution in left tectum (normal retinal input) and right tectum (no retinal input) of left eye-enucleated larvae. *N* = 26 and 19 neurons from 9 larvae for each condition, two-way ANOVA, interaction effect *p*=0.985, with posthoc unpaired *t* test. **H**, **I** Comparison of response onset (LUX at half max) and response duration (LUX at half max) in the left and right tectum of left eye-enucleated larvae presented with 10s ramp stimuli. Two-way ANOVA, interaction effects *p*=0.607 and 0.946, with posthoc unpaired *t* test. Note: for *t* test comparisons in **G**–**I** only *p* values that reached significance are shown. Scale bar: 50μm in **B**, **C,** and **E**

In larval zebrafish the retinal projection to tectum is entirely crossed (Fig. 1), suggesting that monocular stimulation of the right eye should only activate PyrNs in the left (contralateral) tectum. During our initial characterization of PyrN responses to luminance ramp stimuli, we consistently observed active PyrNs in both tectal lobes of every larva examined. To quantify binocular responses in PyrNs, we used two laterally positioned displays to sequentially present monocular stimuli to each eye while imaging the same PyrNs (Fig. 4A). In every larva examined, both tecta contained neurons that responded to ramp stimulus presentation to either eye (Fig. 4B, C). Overall, 81.7±3.85% of active neurons exhibited binocular responses (61 of 74 PyrNs imaged in 6 larvae). Although the projector screen was positioned laterally and the imaging enclosure is constructed of non-reflective materials, it remained possible that responses in ipsilateral tectum could be due to stray stimulus light reaching the contralateral eye. To exclude this, we conducted experiments on larvae in which the left eye was surgically removed at 3 dpf and the intact right eye was visually stimulated at 7 or 8 dpf (Fig. 4D; Additional file 7: Data S5). These enucleated larvae exhibited similar numbers of active PyrNs in both the left/innervated and right/deinnervated tectum (Fig. 4E). Furthermore, PyrN responses in the deinnervated tectum exhibited ramp-evoked responses that were indistinguishable from those in the normally innervated tectum (Fig. 4F). Identification of PyrN responses using the SVM classifier revealed no significant difference in the proportion of ON, DUAL, and OFF classes between the innervated and deinnervated tectum (Fig. 4G). Overall, response kinetics were similar within each class for LUX at half-max and width at half-max in left/innervated and right/deinnervated tectum (Fig. 4H, I). However, OFF responses were observed to have a slightly longer width at half-max in the deinnervated tectum (Fig. 4I, *p*=0.0009, unpaired *t* test). Together these findings suggest that PyrN responses elicited by ipsilateral eye stimulation arise via interocular transfer from the contralateral to ipsilateral (deinnervated) tectum.

### Torus longitudinalis is the site of binocular integration

In zebrafish larvae, the retinal projection is completely crossed (Fig. 1); therefore, the ipsilateral eye cannot drive responses in PyrNs devoid of contralateral retinal input. Although TL does not receive direct retinal input, it receives visual inputs indirectly via inputs from tectum and pretectum [9, 17]. We hypothesized that TL input to tectum may drive visual responses in PyrNs devoid of retinal input. Given that PyrNs lacking retinal input exhibited normal responses to ramp stimuli, we predicted that TL neurons that form feedback projections to tectum exhibit ON, OFF, and DUAL ramp-evoked responses. One way that visual information reaches TL is via TLPNs located in tectum (Fig. 1). We recently characterized TLPN OFF responses to light steps [23]. Although TLPNs represent only 11.8% of labeled neurons in the *id2b:gal4* transgenic, we were able to record specifically from TLPNs by imaging their axons within TL (Additional file 8: Figure S3). Ramp-evoked responses in TLPN axons indeed consisted of ON, OFF, and DUAL classes (16.3%, 80.3%, and 3.3% of 61 ROIs in 12 larvae), with OFF responses representing a large majority. This biased distribution helps explain why our initial characterization of TLPN responses identified only dimming-responsive TLPNs [23]. DUAL responses were rarely observed in the TLPN population labeled by the *id2b:gal4* transgenic (3.3% of 61 ROIs). It is unclear whether this is due to sparse DUAL input to TL or preferential labeling of ON and OFF TLPNs by the *id2b:*gal4 transgene. However, the presence of ON, OFF, and DUAL response classes within TLPNs that provide input to TL is consistent with TL neurons exhibiting similar visual responses to those in PyrNs.

**Fig. 5:**
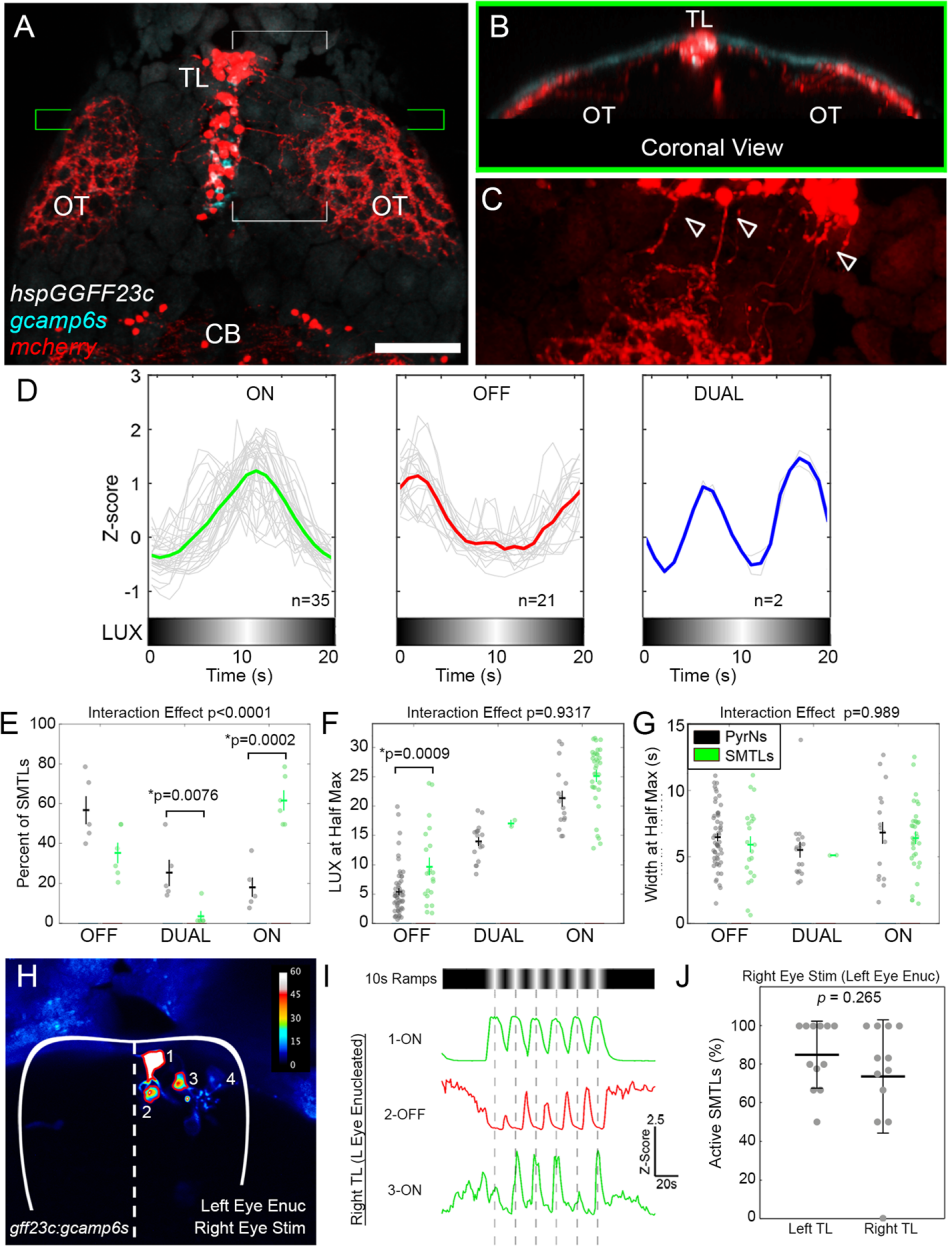
TL is the site of binocular integration. **A** Maximum projection image acquired from a 7dpf Tg(*hspGGFF23C,uas:gcamp6s,uas:mcherry)* triple transgenic larva. Note labeled cell bodies in TL and axonal plexus in OT. **B** Coronal view of green bracketed region in **A**. Note that axons from TL target the SM layer, directly beneath the skin overlying the tectum. **C** Magnified view of white bracketed region in **B** (mcherry channel only). Arrowheads denote SMTL neurons with axons extending into ipsilateral OT. **D** ON, OFF, and DUAL response classes identified in *hspGGFF23C*+ SMTL neurons. **E** Comparison of response class distribution in *id2b*+ PyrNs and *hspGGFF23C*+ SMTL neurons. Note greater proportion of ON responsive units among *hspGGFF23C*+ TL neurons. Two-way ANOVA, interaction effect *p*<0.0001, with posthoc unpaired *t* tests. **F** Comparison of response onset (LUX at half-max) in PyrNs and SMTLs in response to 10s ramp stimulus. Two-way ANOVA, interaction effect *p*=0.9317, with posthoc unpaired *t* tests. **G** Comparison of response duration (width at half-max) in PyrNs and SMTLs in response to 10s ramp stimulus. Two-way ANOVA, interaction effect *p*=0.989, with posthoc unpaired *t* tests. Note: for *t* test comparisons in **E**–**G** only *p* values that reached significance are shown. **H** Magnified view of anterior TL in a left eye-enucleated 7dpf Tg(*hspGGFF23C,uas:gcamp6s)* larva. Upon stimulus presentation to the right (intact) eye, three SMTLs in right TL exhibited visual responses (1–3). **I** Responses in SMTL neurons 1–3 in **H** during presentation of 10s ramp stimulus to the right (intact) eye. Note that the three neurons exhibited consistent responses to each ramp cycle. **J** Quantification of percent of SMTLs in left and right TL of enucleated larvae that responded during 10s ramp stimulus presentation to the right (intact) eye. *N* = 12 larvae, paired *t* test. Scale bar: 60μm in **B**, **C**, 30μm in **D**, and 20μm in **K**

To directly examine the response properties of TL neurons that provide feedback inputs to PyrN SM dendrites, we employed *hspGGFF23C,* a Gal4 transgenic that labels SM-projecting TL neurons (SMTLs; Fig. 5A) [22]. SMTLs extend their axons into the ipsilateral tectal neuropil and form a dense neurite plexus within SM [22] (Fig. 5B, C). We monitored ramp stimulus responses in TL of larvae in which the *hspGGFF23C* transgene drove GCaMP6s expression (Fig. 5A). To classify SMTL responses, we employed the linear SVM trained on 10s ramp PyrN data, as used above to classify RGC responses (Additional file 9: Data S6). Similar to the PyrN and RGC populations, the SVM classifier identified ON, OFF, and DUAL SMTL response classes (19 neurons from 6 larvae; Fig. 5D). Compared to PyrNs in tectum, SMTLs exhibited an increase in the proportion of ON responses and a decrease in the proportion of DUAL responses (*p*=0.0002 and *p*=0.0076, unpaired *t* test; Fig. 5E). Furthermore, SMTL OFF responses also had a significant increase in LUX-at-half-max (*p*=0.0009, unpaired *t* test; Fig. 5F). Width-at-half-max measurements between PyrNs and SMTLs revealed no significant differences (Fig. 5G). Responses to 30s ramp stimuli revealed no significant differences in LUX-at-half-max and width-at-half-max between PyrNs and SMTLs (Additional file 6: Figure S2). Overall, the averaged responses in PyrNs, RGCs, and SMTL populations were remarkably similar, particularly for ON and OFF classes (Additional file 10: Figure S4). The presence of ON, OFF, and DUAL ramp-responsive SMTLs with similar response kinetics suggests that TL feedback projections to PyrNs are also class-specific, matched to the visual response properties of each PyrN class.

A conserved feature of TL-tectum circuitry is that PyrNs are innervated by SMTLs located in ipsilateral TL [19–22]. Therefore, if SMTL input drives ramp-evoked responses in PyrNs lacking retinal input, we expected to find SMTLs in ipsilateral (right) TL that respond to stimulation of the ipsilateral (right) eye. The presentation of 10s ramp stimuli sequentially to each eye (as in Fig. 4A) revealed that overall 95.2% (59 of 62 SMTLs from 7 larvae; data not shown) of SMTLs exhibit binocular responses to luminance ramp stimuli. To confirm that SMTL responses in ipsilateral TL are due to interocular transfer of signals originating from ipsilateral retina, we examined responses in left eye-enucleated larvae (as in Fig. 4D). Consistent with our hypothesis, visual signals originating from the right eye were sufficient to drive ramp-evoked responses in ipsilateral/right TL (Fig. 5H). Ipsilateral SMTL responses exhibited the three response types observed in PyrNs: ON, OFF, and DUAL (Fig. 5I and data not shown). Overall, right eye stimulation evoked responses in similar proportions of SMTLs in both contralateral (left) and ipsilateral (right) TL (Fig. 5J; *p*=0.265, unpaired *t* test). This confirms that most SMTLs are activated by ipsilateral eye stimulation, supporting the idea that TL feedback projections drive visual responses in PyrNs lacking retinal input. These findings also identify TL as the site of binocular integration in the TL-PyrN circuit, revealing a novel function for TL in mediating interocular transfer of luminance information.

### Loss of TL input reduces binocular responses in PyrNs

**Fig. 6:**
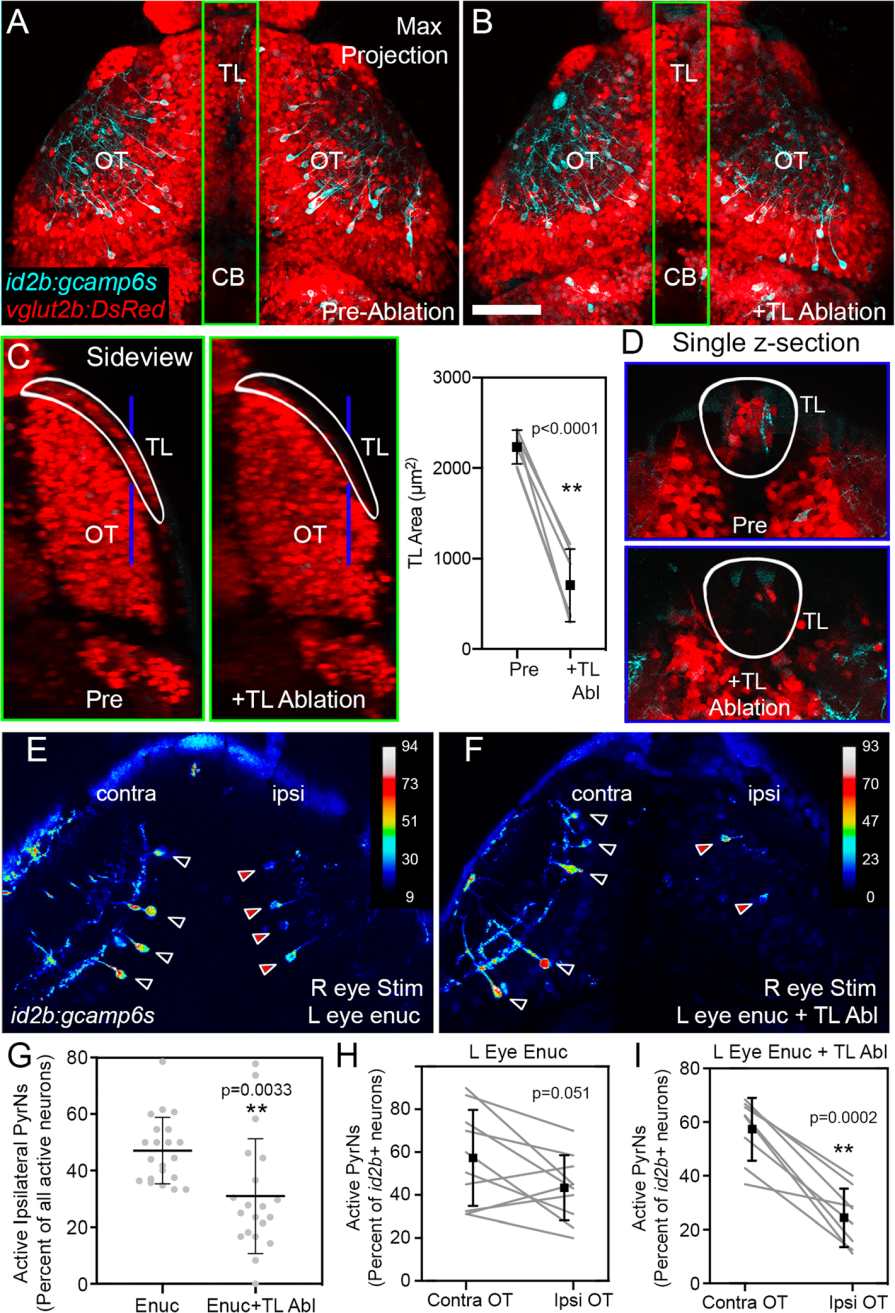
TL is required for PyrN binocularity. **A** Maximum projection image of a 6 dpf *Tg*(*vglut2a:dsred, id2b:gcamp6s)* double transgenic larva prior to laser ablation of TL. **B** Larva in **A** at 7dpf, 1 day following targeted laser ablation of TL at 6 dpf. Note normal brain structure and similar density of GCaMP6s+ PyrNs in OT. **C** Sideview images of green-boxed regions in **A** and **B**. Note reduction in *vglut2a:dsred*+ cells in TL ablated larva. Graph at right depicts quantification of TL ablation using area measurements obtained from sideview projections. *N* = 6 larvae, paired *t* test. **D** Single confocal images at *Z*-planes through dorsal TL indicated by blue lines in **C**. Note strong reduction in *vglut2a:dsred*+ cells in TL ablated larva. **E** StDev projection image of timeseries acquired from a left eye enucleated larva with intact TL. Black arrowheads denote active neurons in contralateral OT with intact retinal input, red arrowheads denote active neurons in ipsilateral OT devoid of retinal input. Note similar proportion of active PyrNs in contra and ipsi OT. **F** StDev projection image of timeseries acquired from a left eye enucleated and TL-ablated larva. Black arrowheads denote active neurons in contralateral OT with intact retinal input, red arrowheads denote active neurons in ipsilateral OT devoid of retinal input. Note reduced number of active neurons in ipsilateral OT compared to contra OT. **G** Percentage of active neurons located in right/ipsilateral tectum during stimulation of right eye in enucleated larvae and larvae that underwent both enucleation and TL ablation. *n*=21 and 20 larvae, *p*=0.0033, unpaired *t* test. **H** Quantification of active PyrNs (expressed as a percentage of total id2b+ neurons) in ipsilateral vs. contralateral tectum in left eye-enucleated larvae during 10s ramp presentation to right eye. *N*=10 larvae, paired *t* test. **I** Quantification of active PyrNs (expressed as a percentage of total id2b+ neurons) in ipsilateral vs. contralateral tectum in larvae that underwent both enucleation and TL ablation during 10s ramp presentation to right eye. Note strong reduction in active PyrNs in all but one larva. *N*=8 larvae, paired *t* test. Scale bar: 75μm in **A**–**B**, 50μm in **C**–**D**, and 60μm in **E**–**F**

To test the necessity of TL input for interocular transfer to PyrNs, we developed a protocol for targeted TL laser ablation. We utilized triple transgenic *vglut2a:dsred,id2b:gal4,uas:gcamp6s* larvae, in which most glutamatergic neurons are labeled with the red fluorescent protein DsRed [28]. Both tectum and TL contain a large proportion of glutamatergic neurons (Fig. 6A–D). One challenge in developing this protocol was that TL is a curved structure that wraps around anterior tectum (Fig. 6C), necessitating multiple, high energy laser scans at multiple depths. During initial optimization, we found that survival was reduced when more than 10 laser ablations were performed in a single larva, possibly due to tissue damage or accumulation of free radicals. However, in larvae that underwent 8–10 ablation scans, survival rates were high and larvae exhibited normal swimming behavior. This many laser ablation scans targeting TL caused no visible change in overall brain structure or distribution of *vglut2a*- or *id2b*-positive neurons in the tectum (Fig. 6A, B). Side-view maximum projections were used to estimate TL volume changes caused by laser ablation (Fig. 6C). In a group of larvae examined pre- and post-TL ablation, we observed a significant 68.9±6.7% reduction in TL area in side-view projections (Fig. 6C, right panel; *n* of 6 larvae, *p*<0.0001, paired *t* test). Although loss of TL was not complete, the dorsal portion of TL containing most TL projections and many visually responsive neurons [22, 23] was effectively ablated using this protocol (Fig. 6D).

To test whether TL is required for transfer of visual information from contralateral tectum to ipsilateral PyrNs, we performed TL laser ablations on *vglut2a:dsred,id2b:gal4,uas:gcamp6s* larvae enucleated at 3–4dpf. As a control, a similar number of larvae were enucleated but not subjected to laser ablation. Following a 1-day recovery from laser ablation, 10s luminance ramp stimuli were presented to the intact eye and active PyrNs were detected in both contralateral and ipsilateral tectum (Fig. 6E, F). In most larvae, we were unable to obtain accurate counts of inactive PyrNs due to their low level of fluorescence. Therefore, in 41 larvae, we quantified the number of active PyrNs in ipsilateral tectum and expressed this value as a percentage of all active PyrNs (ipsilateral + contralateral). In enucleated larvae, 47.1% of active neurons were located in the deinnervated ipsilateral tectum (Fig. 6E, G). Combining enucleation with TL laser ablation led to a significant reduction in the percentage of active PyrNs in ipsilateral tectum (Fig. 6F, G). In a subset of larvae with bright GCaMP6s expression (10 enucleated and 8 enucleated + ablated), we were also able to count inactive PyrNs, permitting active PyrNs to be expressed as a percentage of all GCamp6s-positive neurons in each tectum. In this dataset, enucleation alone did not strongly reduce the percentage of active PyrNs in the ipsilateral tectum compared to contralateral tectum (Fig. 6H; *p*=0.051, paired *t* test). Pairing enucleation with TL ablation led to a significant decrease in the percentage of active PyrNs in ipsilateral tectum compared to contralateral tectum (Fig. 6I; *p*=0.0002, paired *t* test). The partial reductions observed are most likely due to incomplete TL ablation. However, these data do support a direct role for TL inputs in driving binocular responses in PyrNs.

### PyrNs have complex receptive fields that span a large portion of the visual field

**Fig. 7:**
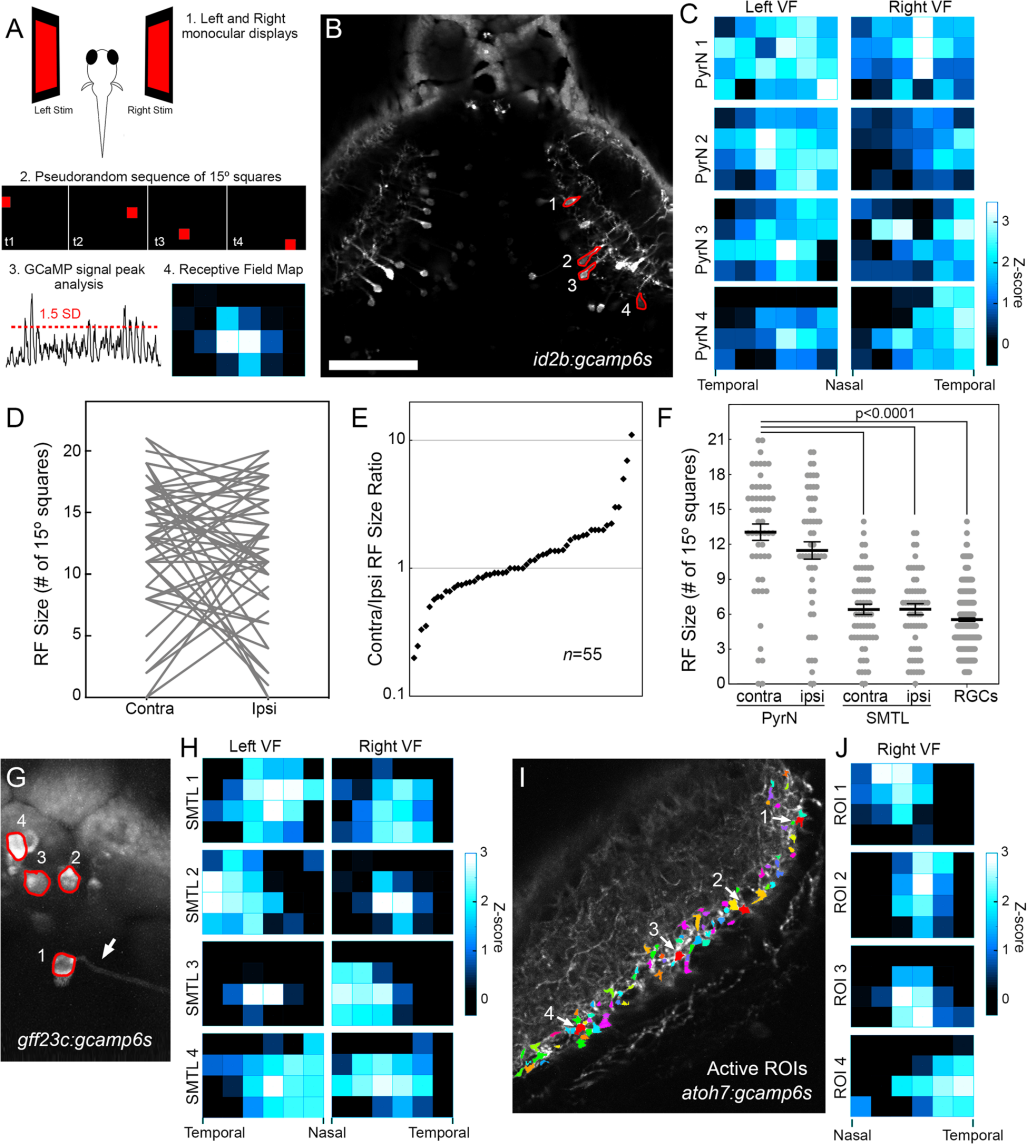
Visual receptive fields of PyrNs, SMTLs, and RGCs. **A** Overview of experimental design to monitor ramp-evoked responses in elements of the tectum-TL circuit. Left and right displays are used to present single 15° squares within a 6x4 checkerboard in a pseudorandom pattern. GCaMP6s signal intensity was used to quantify response strength at each position. Position response strength was subsequently used to generate RF maps. **B** Maximum projection image of an image timeseries acquired in tectum of a *id2b:gcamp6s* larva presented with RF mapping stimuli. **C** Example RF maps for the four PyrNs indicated in **B**. Note large, complex RFs and asymmetries in RF size between left and right visual fields. **D** Quantification of RF size in contralateral and ipsilateral RFs of individual PyrNs. Note high degree of variability in RF size and contra/ipsi ratio. **E** Distribution of contra/ipsi RF size ratio for 55 PyrNs recorded in 7 larvae. **F** Comparison of RF size in PyrNs, SMTLs, and RGCs. One-way ANOVA with Tukey’s multiple comparisons test, *p*<0.0001 for comparisons between each indicated group and the contra-PyrN group. **G** Maximum projection image of an image timeseries acquired in TL of a *gff23c:gcamp6s* larva presented with RF mapping stimuli. Arrow denotes SMTL with visible axon extending towards the right tectal lobe. **H** Example RF maps for the four SMTLs indicated in G. Note large, but discrete RFs and asymmetries in RF size between left and right visual fields. **I** Maximum projection image of an image timeseries acquired in the left tectum of a *atoh7:gcamp6s* larva presented with RF mapping stimulus to the right eye. Overlaid on this image are active RGC ROIs detected in response to presentation of a 30s luminance ramp stimulus to the right eye. Arrows denote four RGC ROIs at distinct positions along the **A**–**P** axis. **J** Example RF maps for the four RGC ROIs indicated in **I**. Note compact, discrete RFs and nasal-temporal distribution of RF positions match anteroposterior position of ROIs in the tectum. Scale bar: 75μm in **B**, 25μm in **G**, and 40μm in **I**

The above results predict that PyrNs have large visual RFs that span both retinal hemifields. To directly test this hypothesis, we designed a protocol to sequentially map PyrN RFs in each retinal hemifield. Two separate displays were positioned to each side of the larva to present RF mapping stimuli to each eye sequentially. Each display spanned 90° of the visual field horizontally and 60° vertically (Fig. 7A). RF mapping stimuli consisted of a sequence of 15° squares, presented in a pseudorandom pattern, at each position of a 6 x 4 array. Each square was presented for 10s with an interval of 10s between each presentation. To prevent eye movements during RF mapping, larvae were paralyzed using intraspinal injection of alpha-bungarotoxin prior to imaging. Under these conditions, we never observed eye vergence, ensuring that our laterally positioned displays did not overlap with the larval binocular zone [29]. The peak GCaMP6s signal during each stimulus presentation was used to generate RF maps and calculate RF size (Fig. 7A). This analysis revealed that PyrN RFs were large and often spanned a large fraction of each display (Fig. 7B, C). Often, these large RFs appeared comprised of multiple, discontinuous regions intermingled with regions that evoked weaker responses (for example see Fig. 7C PyrN1-Left visual field and PyrN3-Right visual field). Consistent with our previous finding that PyrNs are binocular, the vast majority of PyrNs (50 of 54) possessed a RF comprised of regions in the contralateral and ipsilateral retinal hemifields (Fig. 7C). To calculate the size of these irregularly shaped PyrN RF maps, we counted the number of stimulus positions that evoked a GCaMP6s signal response with a *Z*-score ≥ 1.5 (1.5 SDs above the mean for the entire trace). In binocular PyrNs, RFs were often asymmetrical, with the RF in one visual hemifield being larger than the other. The larger RF could be located in either the contralateral or ipsilateral visual field (Fig. 7D), although the majority of PyrNs had similarly sized RFs in both visual fields, as evidenced by contralateral/ipsilateral RF size ratios between 0.5 and 2 (41 of 55; Fig. 7E). Although there is a well described retinotopic map in the tectum, we did not observe an obvious relationship between PyrN cell body position in the tectum and the location of either contra or ipsi RFs. There also did not appear to be a strong correlation between the location of contra and ipsi RFs for individual PyrNs. These findings suggest that one function of the TL-tectum circuit is to generate complex PyrNs RFs that span large portions of the visual field.

To examine spatial summation of RFs at successive stages in the TL-PyrN circuit, we additionally performed RF mapping in both SMTLs and RGCs. Using the *hspGGFF23c* transgenic to drive GCaMP6s expression in SMTLs, we found that the majority of SMTL neurons had RFs that included regions of both retinal hemifields (56 of 59; Fig. 7G, H), additional evidence that binocular integration occurs in TL. Similar to PyrNs, the majority of SMTLs had contralateral/ipsilateral RF size ratios between 0.5 and 2 (data not shown). However, contralateral and ipsilateral SMTL RFs were significantly smaller than those observed in PyrNs (Fig. 7F). Similar to PyrNs, we did not observe a strong correlation between the location of contralateral and ipsilateral RFs in SMTLs. There was also no obvious correlation between SMTL position and RF location, as evidenced by the varied RF sizes and locations observed in the four closely situated SMTLs shown in Fig. 7G, H. To map RFs in RGCs, the *atoh7:gal4* transgenic was used to drive GCaMP6s expression in all RGCs (Fig. 7I). We restricted our analysis to RGCs likely to provide synaptic input to PyrNs by presenting a 30-s luminance ramp stimulus prior to the RF mapping stimulus. This approach allowed us to selectively map RFs in RGC terminals with strong ramp-evoked responses (Fig. 7I). RFs in ramp-responsive RGCs were smaller than those in PyrNs (Fig. 7F), though not significantly smaller than SMTL RFs (*p*=0.3790, one-way ANOVA with Tukey’s multiple comparisons test). In addition, RFs in RGCs exhibited clear retinotopic topography, with ROIs in anterior tectum typically having RFs located in the nasal half of the contralateral visual field and ROIs in posterior tectum typically having RFs located in the temporal half (Fig. 7J). These data support our model in which spatial summation of visual RFs in the TL-tectum pathway is driven by convergent TL input onto PyrNs.

## Discussion

Our findings identify a role for the zebrafish TL in mediating interocular transfer of visual information between the two lobes of tectum. Binocular integration in this circuit first occurs in TL and binocular responses in PyrNs require synaptic input from SMTLs. The TL imparting tectal neurons with binocularity is a unique function for a higher order visual brain area. Binocular neurons are present in the tectum of birds, frogs, and fish [30–32]. Although the larval zebrafish optic tract is completely crossed, circuits in pretectum dedicated to processing whole-field motion contain binocular neurons [33–35]. Binocular integration in these circuits is most likely generated by commissural interneurons connecting both sides of pretectum [36, 37]. Transfer of visual information between the two lobes of tectum has been shown to be mediated by a population of intertectal neurons [38]. These intertectal neurons were shown to be selective for prey-like visual stimuli, suggesting that different types of visual information are transmitted between tecta via dedicated circuits. In the TL-PyrN circuit, we have demonstrated that binocular integration occurs in TL (Fig. 6) and may arise via commissural axons of TLPNs that enter TL and cross the midline to innervate contralateral TL [9]. However, only 30% of TLPN axons are commissural, suggesting that local TL circuits may also play a significant role in binocular integration.

**Fig. 8:**
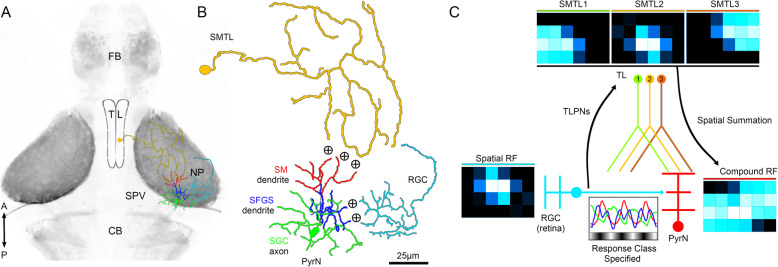
Model for spatial summation in the TL-PyrN circuit. **A** Dorsal view image of a *Tg(HuC:lynTagRFP-t)* larval brain. In this transgenic, all axon tracts and neuropil areas are fluorescently labeled. Image grayscale was inverted for clarity. Overlayed on the midbrain are reconstructions of a single SMTL (orange), a single RGC axon (cyan), and a single PyrN (red, blue, and green). **B** Magnified view of SMTL, RGC, and PyrN morphologies. Note large, sparsely branched SMTL axon that forms excitatory inputs (+) onto the SM PyrN dendrite (red) and the compact RGC axon that forms excitatory input (+) onto the SFGS PyrN dendrite (blue). **C** Model of how spatial summation in the TL-tectal circuit could be mediated by neural convergence at the SMTL-PyrN synapse. Direct RGC input to PyrNs specifies response class. In parallel, the same RGCs provide input to TLPNs that drive visual responses in TL. Three SMTLs with intermediate sized RFs converge onto the same dendrite. Spatial summation of inputs with partially overlapping RFs results in a large, compound RFs in PyrNs

We also provide evidence that spatial summation in the TL-tectum circuit generates the compound visual RFs observed in PyrNs. Tectal neurons with compound visual RFs have been previously described in adult goldfish and zebrafish [6, 39], suggesting this feature is conserved among teleosts. Binocular RF mapping in three different transgenics (*id2b:gal4, hspGGFF23C*, and *atoh7:gal4*) directly demonstrated that PyrN Visual RFs are far larger than those of either input neuron population (SMTLs or RGCs; Fig. 7). Several lines of evidence suggest that spatial summation in this circuit is driven by neural convergence at the TL-PyrN synapse. Anatomical evidence for this has been provided by our previous findings that (1) SMTL axons in SM layer of tectum are extremely large and exhibit a high degree of overlap and (2) PyrN SM dendrites are small and densely innervated [22]. This combination of pre- and postsynaptic morphologies creates a scenario where it is feasible for many SMTLs to synapse onto each PyrN. Our functional RF mapping data supports a model in which highly convergent TL input drives spatial summation to generate large, compound visual RFs in PyrNs. An additional feature of this indirect visual pathway to PyrNs (RGC-TLPN-SMTL-PyrN) is that light level information is relayed without obvious changes in response kinetics or response class diversification. Together these findings suggest that the direct visual pathway to PyrNs (RGC-PyrN) specifies PyrN response class (ON, OFF, DUAL; Fig. 8) whereas the indirect pathway (RGC-TLPN-SMTL-PyrN) expands PyrN RFs.

Although our data support distinct roles for retinal and TL input to PyrNs, it should be noted that the *id2b:gal4* transgene does not label PyrNs exclusively, although they are the majority (73.5%). In preliminary trials, we found that TGPNs, which comprise 14.7% of neurons labeled by *id2b:gal4*, do not respond to whole-field stimuli (data not shown) and therefore would most likely be excluded as inactive neurons in datasets examining responses to luminance ramp stimuli (Fig. 2G). Anatomically restricting our analysis to cell bodies located in the neuropil or shallow SPV was an additional way to ensure TGPN exclusion from our datasets. Therefore, we estimate that our dataset likely consisted of 86% PyrNs and 14% TLPNs. Anatomically targeted recordings from axons of TLPNs during luminance ramp presentation revealed ON, OFF, and DUAL responses similar to those in PyrNs, RGCs, and SMTLs (Additional file 8: Figure S3). Therefore, TLPNs were most likely a small fraction of the ramp-responsive *id2b:gal4*-positive neurons identified in our datasets. Inclusion of TLPNs could explain the 18% of *id2b:gal4*-positive neurons that were monocular, although we cannot exclude the possibility that a fraction of PyrNs are also monocular. In summary, TLPN contamination in our datasets is likely to have slightly inflated counts of active PyrNs in the *id2b:gal4* transgenic and subtly altered response class proportions. On the other hand, the fact that TLPN responses were indistinguishable from PyrN responses (Additional file 10: Figure S4) is consistent with a visual relay from ramp-responsive RGCs to TL via specific classes of TLPNs.

A notable feature of this system is that light level fluctuations are encoded by three distinct response types, not merely ON and OFF types. A third type of response encodes both increases and decreases, albeit within different light intensity ranges than ON or OFF neurons (Fig. 2). PyrN responses are reminiscent of luminance-sensitive neurons in V1 and V2 areas of macaque cortex during presentation of slow luminance oscillations [40]. This study identified neurons with spike rates that mirrored the luminance oscillation (analogous to ON PyrNs), neurons that responded during the trough of the oscillation (analogous to OFF PyrNs), and a third response type termed “gray-preferring neurons”. These neurons responded to luminance oscillations with two peaks, one during the decreasing phase and the other during the increasing phase, similar to DUAL PyrN responses. Combinatorial encoding of luminance oscillations, in which specific luminance ranges are encoded by distinct response types, was termed “peaked encoding” [40]. One advantage of this strategy is that intermediate light intensities are encoded by the activity of DUAL/gray-preferring neurons, whereas in a system containing only ON and OFF neurons intermediate light values would need to be deduced from the relative activity levels of ON and OFF neurons. One difference between these two studies is that OFF neurons in macaque cortex exhibited very brief increases in firing rate (100–200 ms) during the trough of 5 s luminance oscillations. This is in contrast to OFF responses in our system, which had durations of 5–6 s during luminance ramps with a 20s cycle time. One reason for this could be that our use of a calcium indicator with slow off kinetics (GCaMP6s) artificially increased response durations. Future experiments utilizing targeted PyrN electrophysiology or imaging with fluorescent voltage sensors will be required to directly examine this possibility.

Visual neurons specialized to encoding gradual changes in scene luminance may seem odd considering the retina’s ability to rapidly adapt to illumination level [41]. During presentation of slow ramp stimuli, one might predict that most RGCs would rapidly adapt by normalizing their baseline firing rate—a process thought to occur within 250ms [42]. One possibility is that RGCs encoding gradual changes in luminance may lack rapid adaptation mechanisms. One such class of RGCs are melanopsin-expressing intrinsically photosensitive RGCs (ipRGCs) [43, 44]. Although ipRGCs receive synaptic input from rod- and cone-driven retinal circuits, they lack rapid adaptation mechanisms found in conventional RGCs [44, 45]. Slower spike rate adaptation allows ipRGCs to generate sustained responses to hours-long light steps [44]. Our findings suggest that zebrafish RGCs providing input to PyrNs and TLPNs encode changes on shorter timescales than ipRGCs (tens of seconds vs. hours) but resemble them in lacking inputs that drive rapid adaptation. Another similarity between the RGCs we identified and ipRGCs is responsiveness to whole-field stimuli. This is most likely due to lack of an inhibitory surround, as demonstrated in both primate [46] and mouse [47] ipRGCs. These reports are consistent with our finding that RGCs that respond strongly to whole-field ramp stimuli can have relatively small RFs (Fig. 7). Despite these similarities, we do not believe the RGCs identified in our study are ipRGCs, as all ipRGCs reported to date are ON responsive and zebrafish ipRGCs innervate the SAC/SPV layer of tectum [48] and not SFGS. The most likely explanation is that these ON, OFF, and DUAL RGCs are downstream of photoreceptors yet share functional properties with ipRGCs that enable encoding of gradual changes in light level.

A role for TL feedback in expanding PyrN RFs contrasts findings in the mammalian cortex, where feedback projections from higher-order visual areas function to sharpen the visual RFs of neurons in primary visual cortex (V1) [49]. However, feedback projections from the mouse lateromedial visual area to specific V1 interneuron types have recently been shown to generate compound RFs that contain antagonistic feedforward- and feedback-driven regions [50]. Spatial summation driven by feedback projections may be a conserved strategy for generating neurons responsive to uniform changes in the visual scene. One important distinction between that study and our current findings is that that feedforward (retina-driven) and feedback (TL-driven) inputs to PyrN circuit are cooperative, summing to generate large, compound RFs.

In larval zebrafish, the tectum is also reciprocally connected with nucleus isthmi (NI) [51, 52], another second-order visual area not innervated by RGCs. A role for NI in larval zebrafish hunting behavior has recently been described [51]. This study found that a subset of cholinergic NI neurons project to tectum, respond to prey-like stimuli, and are required for normal hunting efficiency [51]. These results suggest that in part of the NI-tectum circuit visual acuity is preserved via topographically precise feedback projections. This is in contrast to our finding that TL feedback projections to PyrNs relay light level information while degrading spatial precision (Figs. 7 and 8). However, in this study, the zebrafish NI was also found to contain a second population of cholinergic tectum-projecting neurons that responded to whole-field contrast steps. Interestingly, many of these neurons bilaterally innervate the tectum and their axons stratify within the SGC layer of tectum [51], the same layer in which PyrNs form a mixed axonal/dendritic arbor [22]. However, it is unclear if these NI inputs and local PyrN activation serve a similar, possibly redundant role in elevating tectal ACh levels in response to luminance changes. Alternatively, NI inputs could synapse directly onto the PyrN SGC dendrite to modulate its visual responses.

Overall, we favor a model in which the TL-PyrN circuit serves a general role in priming tectal circuitry in response to a dynamic visual environment. How could changes in visual scene statistics be transformed into a neural signal that boosts tectal sensitivity? In superior colliculus, the mammalian homologue of tectum, visual responses are modulated during saccadic eye movements [53]. One possibility is that scene dynamics alone, irrespective of eye movement, can also modulate visual responses in tectum/SC. A change in mean light intensity within the large, binocular RFs of PyrNs could arise by either (1) a static visual scene undergoing a change in illumination (a scenario mimicked by luminance ramp stimuli) or (2) the retina sampling a region of the external environment with a different ratio of light and dark surfaces. Based on our current findings, we predict that many PyrNs would respond whenever a shift in scene luminance was detected in one or both retinas, with the polarity of the shift determining whether this information was conveyed primarily by ON or OFF PyrNs. We propose that PyrN activity signals that the visual environment is dynamic, boosting the sensitivity of feature-specific tectal circuits that detect ecologically relevant cues such as predators [3, 5] or prey [2, 54]. This enhancement could be mediated by PyrN release of acetylcholine (ACh) and activation of nicotinic ACh receptors (nAChRs) expressed by tectal neurons and RGCs [55]. PyrNs are immunoreactive for ACh synthetic enzymes [9] and their axon is in SGC, a layer that receives RGC inputs. Activation of nAChRs on retinal axons has been shown to enhance retinotectal transmission by depolarizing axon terminals and enhancing neurotransmitter release [56]. PyrN ACh release may also act on SGC-targeted dendrites of tectal neurons to enhance their responses to retinal input. In these scenarios, PyrN ACh release would serve a neuromodulatory role, boosting tectal sensitivity in response to a dynamic visual scene.

## Conclusions

These findings identify a novel role for the zebrafish TL in mediating (1) interocular transfer of visual information between the two lobes of tectum and (2) spatial summation that enlarges the visual RFs of neurons that receive TL input. PyrNs, the postsynaptic target of TL projections to tectum, thereby acquire binocular RFs that span a large portion of the visual field. Several lines of evidence suggest that spatial summation in this circuit is driven by neural convergence at the TL-PyrN synapse. Our functional RF mapping data supports this model, as RF sizes increased incrementally at three subsequent stages of visual processing: retina, TL, and PyrNs. This is the first demonstration of feedback projections from a higher order visual brain area imparting binocularity on first order visual neurons while degrading spatial precision. We propose that this generates a network of neuromodulatory tectal neurons activated in response to a dynamic visual environment.

## Methods

### Transgenic fish

Zebrafish adults and larvae were maintained at 28°C on a 14/10 h light/dark cycle.

*Tg(id2b:Gal4-VP16)mpn215, Tg(UAS-E1B:NTR-mCherry)c264, Tg(hspGGFFgff23c)nk23cEt, Tg(vglut2a:loxP-DsRed-GFP) nns14Tg, Tg(14xUAS:GCaMP6s)mpn101, and Tg(atoh7:gal4)s1992t* transgenic lines have been previously described [25, 28, 54, 57, 58]. All larvae used were double mutants for *mitfa*^*-/-*^ *(nacre)* and *roy*^*-/-*^. All animal procedures conformed to the institutional guidelines of the Purdue University Institutional Animal Care and Use Committee (IACUC).

### Embryo injections

Genetic mosaic labeling of single PyrNs was performed by expression of the membrane targeted EGFP plasmid, 4xnrUAS:mTomato-caax (a gift from B. Appel and J. Hines, University of Colorado, Denver, CO), along with RNA encoding Tol2 transposase into *Tg(14xUAS:GCaMP6s)* transgenic embryos. DNA and RNA mixture at a concentration of 50 ng/μl each was pressure-injected into one- to eight-cell-stage embryos. Embryos were raised in 0.3x Danieau’s solution.

### Enucleations

Three to four dpf larvae were embedded in 2% low-melting-point agarose and anesthetized in 1x Danieau’s solution containing 0.016% tricaine. Using a 27-g syringe needle (Becton Dickinson), the skin overlying the left eye was carefully cut and the optic nerve was cut. The tip of the needle was then used to roll the eye out of the ocular cavity and press the flap of skin over the vacant cavity. Larvae were then released from agarose and placed in 1x Danieau’s solution for recovery overnight. >95% of larvae survived enucleation and exhibited normal swimming patterns.

### Confocal microscopy

For structural analysis, confocal imaging was performed on 6–8 dpf larvae embedded in 2% low-melting-point agarose and anesthetized in 0.016% tricaine (Millipore Sigma). Imaging was performed on a Nikon C2 confocal microscope equipped with solid state lasers for excitation of EGFP (488 nm) and mCherry (555 nm). Whole-brain imaging of live larvae was performed using a Nikon LWD 16x 0.8NA water immersion objective using 1-μm *z*-steps, single-neuron imaging used a Nikon Fluor 60x 1.0NA water immersion objective and 0.375-μm *z*-steps.

### Multiphoton microscopy

GCaMP6s imaging was performed on 7–8 dpf larvae embedded in 2% low-melting-point agarose without anesthetic. For RGC imaging and all receptive field mapping experiments, larvae were first paralyzed by spinal injection of alpha-bungarotoxin (Alomone Labs, Jerusalem, Israel) 30–120 min prior to imaging. Multiphoton imaging was performed on a custom Scientifica (East Sussex, UK) microscope equipped with a Chameleon titanium-sapphire laser (Coherent Inc., Santa Clara, CA, USA) tuned to 920nm for GCaMP6s excitation. All functional imaging was performed using a Nikon LWD 16x 0.8NA water immersion objective. Image acquisition rates were between 1 and 2 Hz. Visual stimuli were generated using PsychoPy software [59] and presented using an ASUS Zenbeam picoprojector (ASUStek, Inc.) equipped with Kodak Red 25 Wratten filter (Edmund Optics, USA).

### Laser ablations

Multiphoton ablation was performed on the same Scientifica microscope used for imaging equipped with a Coherent Chameleon titanium-sapphire laser tuned to 1000nm to image DsRed expression in *vglut2a:loxP-DsRed* larvae, which was used to locate the TL. Following anatomical identification of TL, X-Y subregions at eight *Z*-position were scanned with 800nm laser irradiation at a power of 478mW (measured at the objective). Pixel dwell was 3.8μs and each scan lasted 1 s. 4–8 scans were normally sufficient to ablate TL neurons within the scanning region.

### Image processing

All image stacks were visualized and processed using ImageJ FIJI software (http://fiji.sc/Fiji). In image series targeting PyrNs, TLPNs, and SMTLs, motion artifacts were removed using the StackReg ImageJ plugin [60]. TIFF image files were then processed using background subtraction with a rolling ball radius of 50px. PyrN and SMTL ROIs were manually drawn using maximum projection images of each image stack. In experiments targeting PyrNs, we limited ROI selection to a region of tectum consisting of the deep neuropil and the shallow SPV (see Fig. 2). We estimate that this automatic segmentation of RGC ROIs based on fluorescence activity of pixel clusters was performed using the CalciumSignalExtract GUI for Matlab written by Stephan Meyer [26]. To restrict subsequent analyses to RGCs that responded strongly to ramp stimuli, we chose a detection threshold of 175%, meaning that only pixels with a minimum change of 75% over the average intensity for that pixel were included in the segmentation. These ROIs were then used to analyze RGC responses to different ramp cycle durations.

### Data analysis

All extracted ROI data series were normalized using a *z*-score:

$$ \mathrm{z}=\left(\mathrm{x}\hbox{-} \upmu \right)/\upsigma $$

where *μ* is the mean and *σ* is the standard deviation of the fluorescence from the onset of the stimulus until its end. If a stimulus contained consecutive ramps, the mean response of all ramps was taken as representative of the neuron. Further analysis of individual peak characteristics first included the identification of significant peaks, where a significant peak was defined as having a minimum peak prominence greater than 0.75. For each significant peak, both width and lux at half max were calculated in accordance with the ramp speed and max-lux value of each stimuli. Responses that contained multiple significant peaks, such as in “DUAL” responses, were taken as an average from the width-at-half-max and lux-at-half-max of each significant peak. For classification of active vs inactive neurons in Fig. 6G, H cells were manually selected indiscriminately of perceived activity. Extracted ROI data was normalized using a *z*-score over the stimulus region. Neurons that had no obvious change in fluorescence, where the *z*-score never exceeded the bounds of ±0.5, were flagged as unresponsive. SVM classification analysis was applied to the extracted ROIs, binned altogether regardless of response.

### Linear SVM

A linear SVM model was constructed for each anatomical region and corresponding total stimulus ramp time for a total of six models (PyrNs, RGCs, SMTLs; with either 10s or 30s ramps). SVMs were trained and validated using the Classification Learner from MATLAB’s Statistics and Machine Learning Toolbox version 11.6. Training data consisted of several hundred manually labeled examples of “ON”, “OFF”, “DUAL”, and nonresponsive categories. Models were tested using a 5-folds cross-validation methodology. The best model was selected and saved. SVMs were applied to classify the unlabeled remainder of their respective datasets and visually inspected for accuracy.

### K-means clustering

The optimal number of clusters was determined using the Elbow Method (Sebastien De Landtsheer (2020). kmeans_opt (www.mathworks.com/matlabcentral/fileexchange/65823-kmeans_opt), MATLAB Central File Exchange) with 1000 replicates allowing for a maximum of eight clusters. Seven clusters were identified in every bin of neurons except for 30s ramps in RGCs and 30s ramps in SMTLs where 5 clusters and 6 clusters were identified, respectively. Increasing the limit on the number of clusters only served to generate derivative clusters and reduced the repeatability of the analysis. Clusters containing obviously similar peak characteristics were manually binned into “ON”, “OFF”, “DUAL”, and nonresponsive categories.

### Statistical analysis

Data sets were analyzed using either MATLAB (The Mathworks, Inc. Natick, Massachusetts, USA) or GraphPad Prism software (GraphPad Software, Inc., La Jolla, CA). All data displayed a normal distribution (*p* ≥ 0.05) using the Shapiro-Wilk test. One-way ANOVA was used to identify differences among means for data sets with three or more groups combined with Tukey’s posthoc test for comparisons in Figs. 3D, 5J, and 7F. For these comparisons *p* values less than 0.05 were considered significant. Graphs and table show mean ± SEM for each group, except in Figs. 5J and 6G–I where mean ± SD were shown. When comparing response metrics between two different cell types (e.g., PyrNs, SMTLs, RGCs) as in Fig. 3F–H, Fig. 4G–I, Fig. 5E–G, and supplemental Fig. 2A–F, a two-way ANOVA with a balanced design was used to identify interactions across factors of cell type and response class. First, groups were checked for normalcy using a Shapiro-Wilk test and then Bartlett’s test for homogeny of variance, then an ANOVA was applied. If a significant interaction effect was found (*p* ≥ 0.05), we applied Tukey’s posthoc test to compare response classes across cell types. A Bonferroni correction was applied to these comparisons, such that results were considered significant if the *p* value fell below 0.05/3 = 0.0167.

## Supplementary Information

**Additional file 1: Figure S1.** *K*-means clustering of PyrN responses to ramp stimulus. **A**. K-means clustering output of PyrN responses during 564 cycles of 10s ramps recorded from 94 neurons in 10 larvae. **B**. Averaged responses to 10s ramp stimulus recorded from 94 neurons grouped into ON, OFF, and DUAL classes.

**Additional file 2: Data S1.** Matlab code for linear SVM used to classify responses as On, OFF, or DUAL.

**Additional file 3: Data S2.** Pyramidal neuron calcium responses during presentation of 10s ramp stimulus (xlsx 500kb).

**Additional file 4: Data S3.** Pyramidal neuron calcium responses during presentation of 30s ramp stimulus (xlsx 854kb).

**Additional file 5: Data S4.** RGC calcium responses during presentation of 10s and 30s ramp stimuli (xlsx 742kb).

**Additional file 6: Figure S2.** Summary of PyrN, RGC, and SMTLs response kinetics in response to 30s ramp stimulus. **A.** Comparison of response class proportions, LUX at half-max, and width at half-max for PyrNs and RGC axon ROIs. **B.** Comparison of response class proportions, LUX at half-max, and width at half-max for PyrNs and SMTLs.

**Additional file 7: Data S5.** Pyramidal neuron calcium responses in enucleated larvae during presentation of 10s ramp stimulus (xlsx 871kb).

**Additional file 8: Figure S3.** TLPNs encode gradual changes in illumination with ON, OFF, and DUAL response classes. **A.** Multiphoton image through tectum and TL of an *id2b:gcamp6s* 7dpf larva. Boxed region indicates TLPN axon arbors within TL. **B.** StDev projection image of magnified region indicated by box in A during presentation of 10s ramp stimulus. Note bright axonal varicosities likely to be synaptic boutons. **C.** Active ROIs detected in same image series as B. **D.** TLPN axon responses to 10s ramp stimulus. ON and OFF traces are averaged ± SD of 10 and 49 ROIs, respectively. DUAL trace is a single example of a DUAL TLPN.

**Additional file 9: Data S6.** SMTL calcium responses during presentation of 10s ramp stimulus (xlsx 263kb).

**Additional file 10: Figure S4.** Comparison of response kinetics in PyrNs, RGCs, and SMTLs. **A.** Averaged GCaMPs signal dynamics in response to 10s ramp stimuli for 3 classes of PyrNs, RGCs, and SMTLs. Overlay of traces in fourth row demonstrates the similarity in GCaMP6s dynamics for each functional class (On, OFF, DUAL) across different cell types. **B.** Correlation coefficient matrices for average traces in A. Note large correlation coefficient between cell types for ON and OFF response classes. Also note reduced correlation between DUAL responsive RGCs and SMTLs.

**Additional file 11: Data S7.** SMTL calcium responses during presentation of 30s ramp stimulus (xlsx 378kb).

## Data Availability

All data generated or analyzed during this study are included in this published article, and its supplementary information files and publicly available repositories. Timeseries data analyzed to support the main conclusions of this paper, as well as the MATLAB codes used for this analysis, are included in this published article as supplementary information files. Additionally, raw images and data files will be made available by the authors, without undue reservation, to any researcher through the Purdue University Research Repository (purr.purdue.edu) in a dataset associated with the DOI: 10.4231/STQE-9A91.

## Abbreviations

ipRGCs: Intrinsically photosensitive RGCs
PyrNs: Pyramidal neurons
RF: Receptive field
RGCs: Retinal ganglion cells
SMTLs: SM-projecting TL neurons
SAC: Stratum album centrale
SFGS: Stratum fibrosum et griseum superficiale
SGC: Stratum griseum centrale
SM: Stratum marginale
SO: Stratum opticum
SPV: Stratum periventriculare
SVM: Support vector machine
TGPNs: Tegmental projection neurons
TL: Torus longitudinalis
TLPNs: Torus longitudinalis projection neurons
