# Supplementary figures and images for: Input from torus longitudinalis drives binocularity and spatial summation in zebrafish optic tectum
Source: BMC Biol. 2022 Jan 25;20:24. doi: 10.1186/s12915-021-01222-x (PMC8788132; doi:10.1186/s12915-021-01222-x)

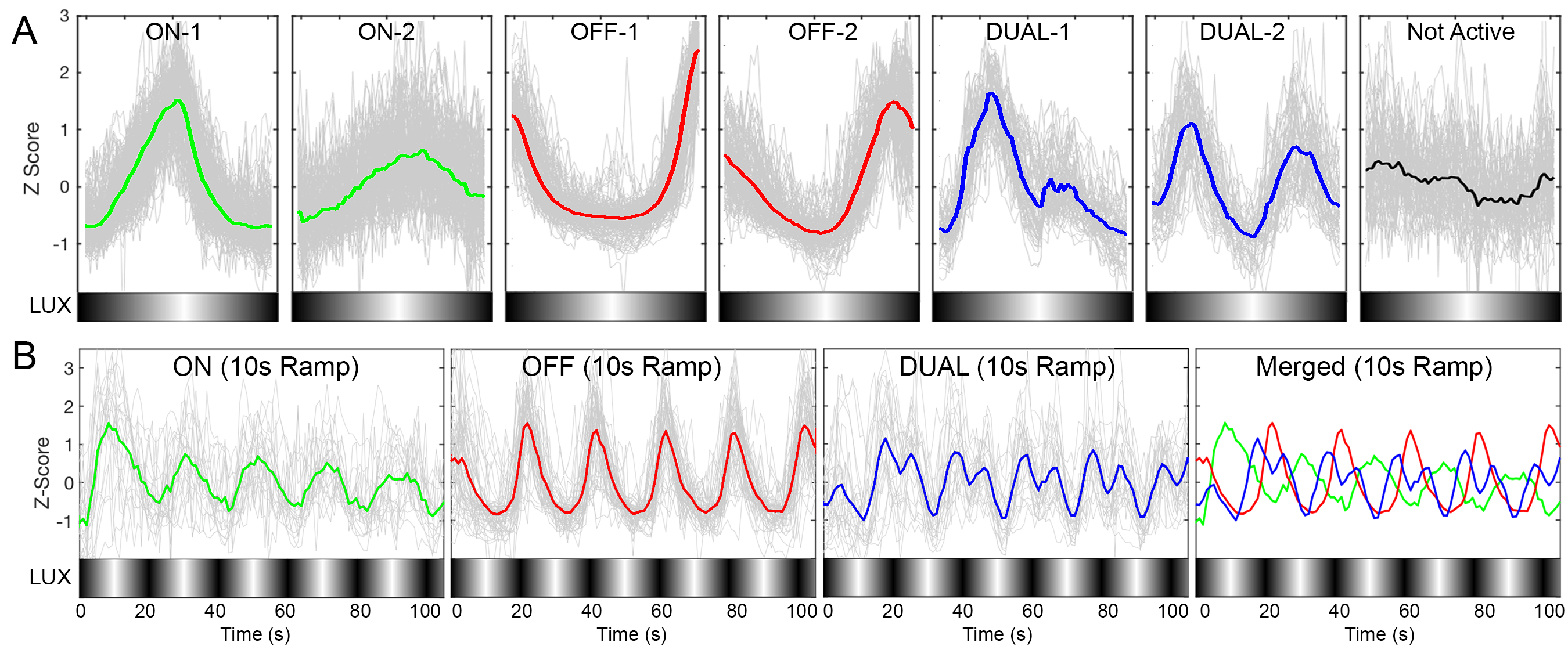

Supplement: Supplementary file 1 — Additional file 1: Figure S1. K-means clustering of PyrN responses to ramp stimulus. A. K-means clustering output of PyrN responses during 564 cycles of 10s ramps recorded from 94 neurons in 10 larvae. B. Averaged responses to 10s ramp stimulus recorded from 94 neurons grouped into ON, OFF, and DUAL classes. [file 12915_2021_1222_MOESM1_ESM.png]

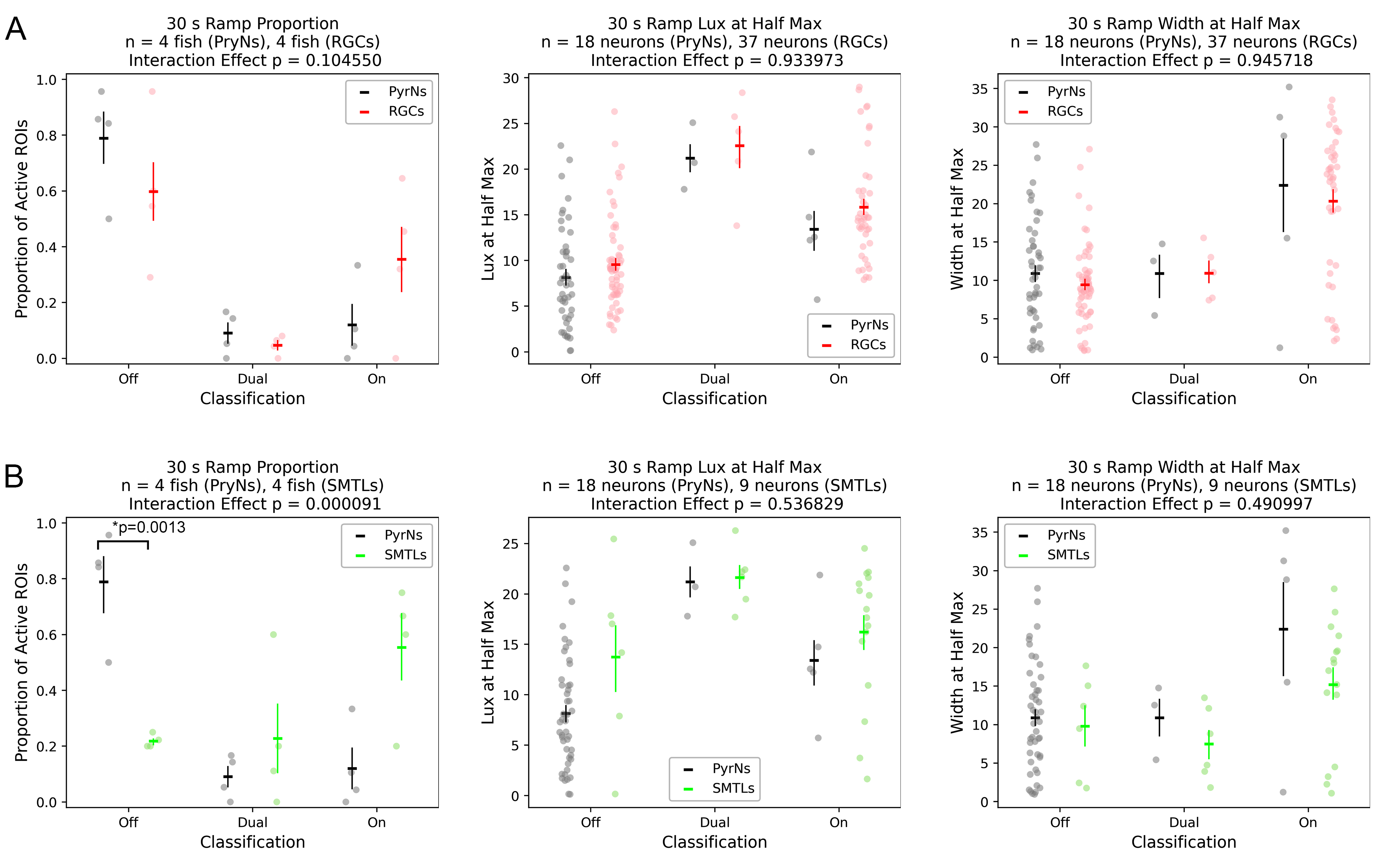

Supplement: Supplementary file 6 — Additional file 6: Figure S2. Summary of PyrN, RGC, and SMTLs response kinetics in response to 30s ramp stimulus. A. Comparison of response class proportions, LUX at half-max, and width at half-max for PyrNs and RGC axon ROIs. B. Comparison of response class proportions, LUX at half-max, and width at half-max for PyrNs and SMTLs. [file 12915_2021_1222_MOESM6_ESM.png]

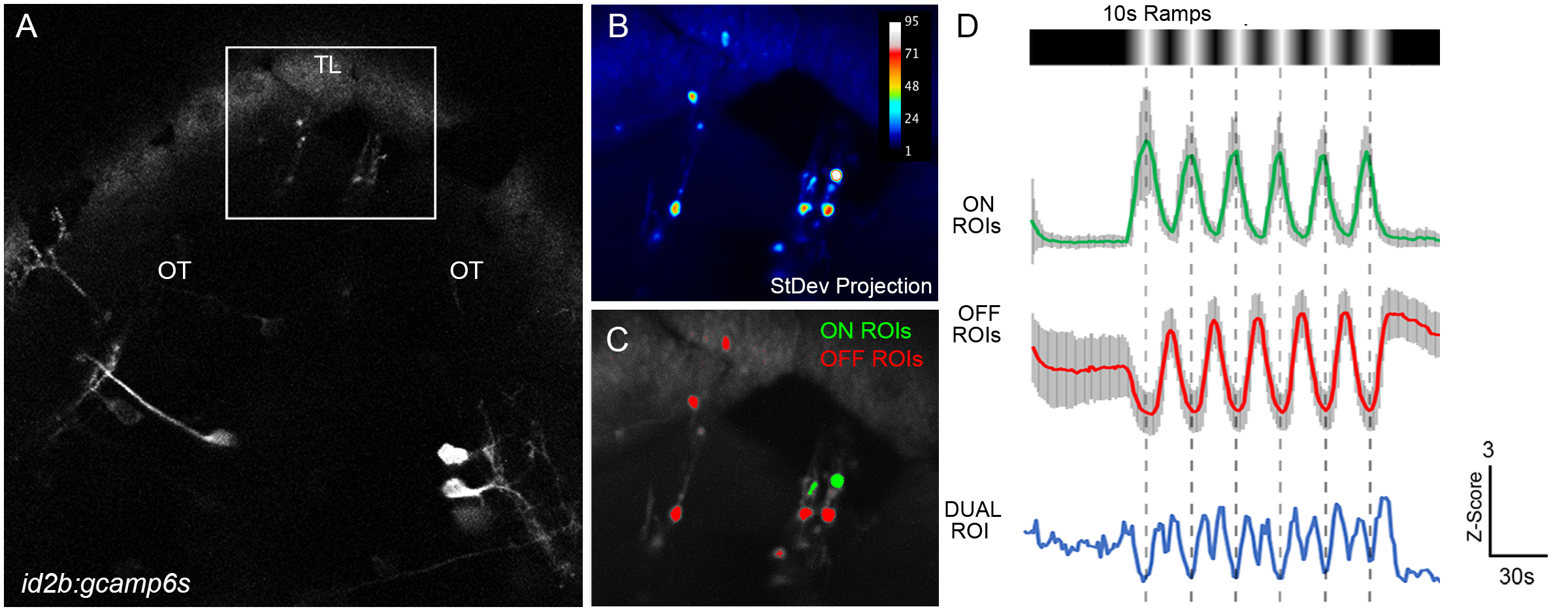

Supplement: Supplementary file 8 — Additional file 8: Figure S3. TLPNs encode gradual changes in illumination with ON, OFF, and DUAL response classes. A. Multiphoton image through tectum and TL of an id2b:gcamp6s 7dpf larva. Boxed region indicates TLPN axon arbors within TL. B. StDev projection image of magnified region indicated by box in A during presentation of 10s ramp stimulus. Note bright axonal varicosities likely to be synaptic boutons. C. Active ROIs detected in same image series as B. D. TLPN axon responses to 10s ramp stimulus. ON and OFF traces are averaged ± SD of 10 and 49 ROIs, respectively. DUAL trace is a single example of a DUAL TLPN. [file 12915_2021_1222_MOESM8_ESM.png]

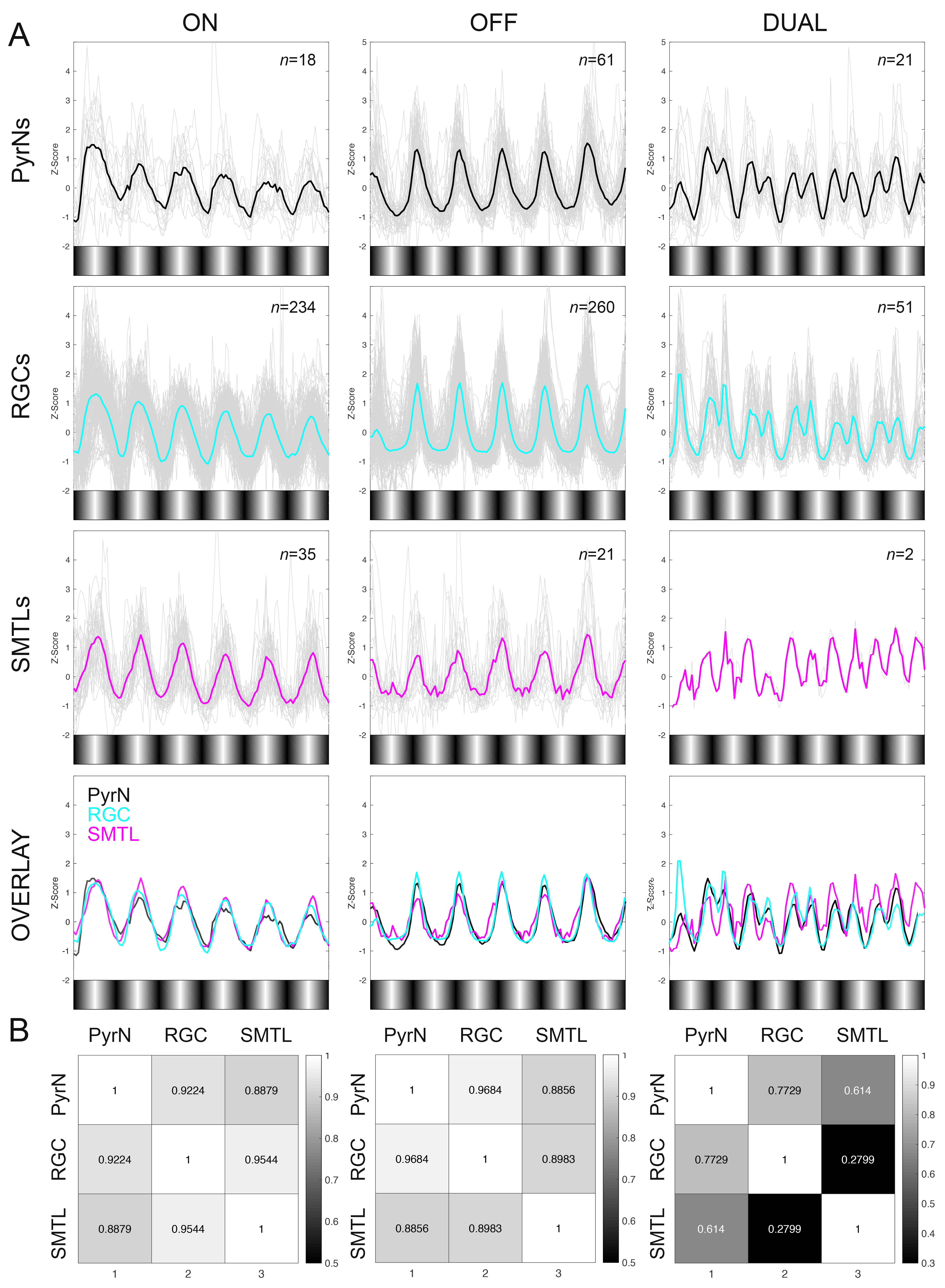

Supplement: Supplementary file 10 — Additional file 10: Figure S4. Comparison of response kinetics in PyrNs, RGCs, and SMTLs. A. Averaged GCaMPs signal dynamics in response to 10s ramp stimuli for 3 classes of PyrNs, RGCs, and SMTLs. Overlay of traces in fourth row demonstrates the similarity in GCaMP6s dynamics for each functional class (On, OFF, DUAL) across different cell types. B. Correlation coefficient matrices for average traces in A. Note large correlation coefficient between cell types for ON and OFF response classes. Also note reduced correlation between DUAL responsive RGCs and SMTLs. [file 12915_2021_1222_MOESM10_ESM.png]
